# Adaptation of lipid metabolism of the polyextremophilic cyanobacterium *Cyanobacterium aponinum* PCC 10605 to adverse environmental conditions

**DOI:** 10.1186/s12870-026-08797-z

**Published:** 2026-04-22

**Authors:** Zishuo Chen, Arpita Shajil Das, Amita Shajil Das, Helga Peisker, Katharina Gutbrod, Georg Hölzl, Gabriel Schaaf, Peter Dörmann

**Affiliations:** 1 Institute of Molecular Physiology and Biotechnology of Plants (IMBIO), University of Bonn, Karlrobert-Kreiten-Str. 13, Bonn, 53115 Germany; 2 Institute of Crop Science and Resource Conservation (INRES), Department of Plant Nutrition, University of Bonn, Karlrobert-Kreiten-Str. 13, Bonn, 53115 Germany

**Keywords:** *Cyanobacterium aponinum*, Triacylglycerol, Membrane Lipid, Multifunctional Acyltransferase

## Abstract

**Background:**

*Cyanobacterium aponinum* PCC 10605 (Cyan10605), a thermophilic, halotolerant single-cell cyanobacterium, can grow under extreme conditions including high temperature and high salinity. While the impact of stress on growth and the production of biotechnologically high-value compounds like exopolysaccharides and phycocyanin have been studied, the responses of lipid metabolism of Cyan10605 under stress conditions remain unclear.

**Results:**

Here, we present the alterations in membrane and nonpolar lipids in Cyan10605 cells during growth under high temperature, salt stress, phosphate and nitrogen deprivation, and extended cultivation time. The degree of acyl unsaturation in all membrane lipid classes decreased at high temperature, while sulfolipids accumulated up to ~ 50 mol% of membrane lipids at the expense of galactolipids in cells grown in artificial seawater medium. The membrane lipid composition under phosphate deprivation remained similar, with low amounts of phosphatidylglycerol, while the proportion of sulfolipid was increased under nitrogen starvation. After extended cultivation, large amounts of membrane lipids were degraded. Triacylglycerol containing saturated acyl groups accumulated at high temperatures, while under phosphate deprivation, the TAG content remained similar, but fatty acid phytyl esters increased. Nitrogen deprivation caused an increase in TAG but a decrease in phytyl esters. After extended cultivation, triacylglycerol and phytyl esters accumulated to the highest amounts. Under all stress conditions, the content of acyl-plastoquinols decreased. Cyan10605 contains a multifunctional acyltransferase related to *Synechocystis* sp. PCC 6803 Slr2103. This enzyme is presumably involved in nonpolar lipid synthesis since it produced TAG and FAPE after expression in *Escherichia coli*.

**Conclusions:**

These results show that membrane lipids and nonpolar lipids are involved in the metabolic response of Cyan10605 to adapt to extreme growth conditions. Small amounts of fatty acids derived from membrane lipids are deposited in the form of TAG and FAPE, but not in acyl-plastoquinols, during extended cultivation, indicating that the synthesis of nonpolar lipid classes is differentially regulated. Knowledge about lipid changes during adaptation to different growth conditions is crucial for biotechnological applications including the use of Cyan10605 for biodiesel production and the optimization of growth in outdoor or mass cultivation settings.

**Supplementary Information:**

The online version contains supplementary material available at 10.1186/s12870-026-08797-z.

## Introduction

Cyanobacteria are prokaryotic organisms that execute oxygenic photosynthesis like plants. The two photosystems, PS II and PS I, in cyanobacteria, green algae and land plants are embedded in thylakoid membranes that consist of only four membrane lipids, two galactolipids (monogalactosyldiacylglycerol, MGDG; digalactosyldiacylglycerol, DGDG), an acidic sulfolipid (sulfoquinovosyldiacylglycerol, SQDG) and an acidic phospholipid (phosphatidylglycerol, PG) [1]. The presence of high amounts of non-phosphorus lipids in the thylakoid membranes represents an adaptive response to phosphate scarcity in many natural habitats. During phosphate deprivation in *Synechococcus* sp. PCC 7942 and other cyanobacteria, the proportion of PG decreases even further, while the amounts of the non-phosphorus lipids DGDG and SQDG increase, thereby releasing phosphate from membrane lipids for DNA and RNA synthesis [2, 3].

Cyanobacterial lipids are considered a promising feedstock for sustainable biodiesel production, and extremophiles are preferred for biotechnological purposes to ease the challenges of large-scale cultivation [4]. We selected the polyextremophilic strain *Cyanobacterium aponinum* PCC 10605 (Cyan10605) to study the lipid responses during different growth conditions. This single-cell cyanobacterium was originally isolated from Euganean thermal springs (Montegrotto, Padua, Italy) [5]. This strain can adapt to freshwater or seawater medium (up to 34 g L^− 1^ NaCl) [5, 6]. The full genome of Cyan10605 was sequenced in 2012 (Genome assembly ASM31767v1; NCBI; JGI Project Id: 1077468). Besides its unique capability of adaptation to various stress conditions, Cyan10605 produces high-value metabolites including exopolysaccharides, phycocyanin, zeaxanthin and β-carotene, which hold a great biotechnological potential as natural colorants and additives for the food industry [7]. Furthermore, Cyan10605 is a promising feedstock for biodiesel production [8]. In addition to Cyan10605, several other related *C. aponinum* strains were described [8–10]. The exopolysaccharides of *C. aponinum* from the thermal spring in the Blue Lagoon, Iceland, have shown immune-modulating and anti-inflammatory effects, which may contribute to the beneficial effects observed in psoriasis patients [11, 12]. Another strain, *C. aponinum* SCSIO-45682, isolated from a marine open pond cultivation system, displayed good adaptation to high alkalinity. It grew optimally in the presence of 8.4 g L^− 1^ NaHCO_3_ (pH 9–10), producing high amounts of polysaccharides and phycocyanin [13, 14].

*C. aponinum* shows a high growth rate at 40 °C and can grow at temperatures up to 50 °C [7, 15]. Saturated and monounsaturated C14 and C16 fatty acids (14:0, 14:1, 16:0, 16:1) are predominant in closely related strains of *C. aponinum* [9, 10]. Previous studies showed that the response of other thermophilic cyanobacteria to elevated temperatures includes a shift from unsaturated fatty acids to saturated fatty acids, accompanied by the increase in the proportion of DGDG relative to MGDG, SQDG and PG [16–18]. However, the lipidomic changes in the polyextremophilic Cyan10605 in response to high temperature and other environmental conditions remain unclear.

Only small amounts of nonpolar lipids have been found in *Synechocystis* sp. PCC 6803, *Synechococcus* sp. PCC 7002 and other cyanobacteria. These lipids include the plastoquinol esters, acyl- plastoquinol-9 (acyl-PQH) and acyl-plastoquinone C (acyl-PQC), triacylglycerol (TAG) and fatty acid phytyl esters (FAPE) [19–22]. A single multifunctional acyltransferase (MFAT) has been implicated in the synthesis of these nonpolar lipids. However, the function of nonpolar lipids under stress conditions remains unclear [19, 21, 22]. Cyan10605 contains one gene (Cyan10605_2185, WP_015219996) with high sequence identity to the MFAT acyltransferase Slr2103 from *Synechocystis* sp. PCC 6803 [19]. Therefore, it is likely that nonpolar lipids are produced in Cyan10605, and they might accumulate in the cells during different stress conditions.

In the present study, we analyzed the impact of different growth conditions, including high temperature, high salinity, phosphate and nitrogen deprivation, and an extended growth period, on the contents of membrane lipids and nonpolar lipids as well as on ultrastructural changes in Cyan10605. Additionally, we conducted heterologous expression of the MFAT from Cyan10605 in *Escherichia coli* and measured the enzymatic activity for nonpolar lipid synthesis. From these experiments it became clear that membrane lipids and nonpolar lipids are involved in the adaptive responses of Cyan10605 to the different stress conditions. The goal of this work was to study the roles of fatty acids, polar and nonpolar lipids in the environmental adaptation of polyextremophilic Cyan10605. This will be crucial to optimize the lipid production for biodiesel application, and to improve the large-scale cultivation conditions of Cyan10605 for biotechnological applications.

## Materials and methods

### Cells and growth conditions

*Cyanobacterium aponinum* PCC 10605 (Cyan10605) cells were obtained from the Pasteur Culture Collection of cyanobacteria (Paris, France). Cells were grown photoautotrophically in modified BG-11 medium (BG-11 supplemented with 0.84 g L^− 1^ sodium bicarbonate) [23]. Starter cultures were maintained in Erlenmeyer flasks at 25 °C with low agitation (100 rpm) and continuous light of 25 µmol photons m^−2^ s^−1^ (measured with a LI-190SB quantum sensor in air attached to a LI-185B quantum/radiometer/photometer in the range of 400–700 nm; LI-Core Inc. USA).

For stress experiments, starter cultures were grown as described above up to an OD_750_ of 0.5. Cells were harvested by centrifugation, washed, suspended in fresh medium and used for inoculation of 150 mL of liquid culture in 250 mL flasks. To investigate the responses of Cyan10605 to high temperature, cells were grown in modified BG-11 medium at 40 °C for 5 days with constant shaking (100 rpm). For salt stress experiments, cells were grown in artificial seawater medium (ASN-III containing 25 g L^− 1^ NaCl, supplemented with 10 µg L^− 1^ vitamin B_12_ and 0.84 g L^− 1^ sodium bicarbonate), and constantly shaken for 10 days. For phosphate deprivation, cells were grown in modified BG-11 medium with a phosphate concentration of 0.04 g L^− 1^ (+ P) or 0 g L^− 1^ (-P) by omitting K_2_HPO_4_, while maintaining the same potassium concentration using KCl. The cultures were grown with constant shaking for 7 days. For nitrogen deprivation, cells were incubated in modified BG-11 medium containing 10 mM NaHCO_3_ without NaNO_3_. The flasks were constantly shaken for 5 days. To study the effect of cultivation time, cultures were incubated in modified BG-11 at 25 °C without shaking for 14 days (control) or 42 days (extended cultivation). Cells were harvested by centrifugation and washed three times with water. The pellets were dried in a SpeedVac evaporator (MAXI dry plus) for dry weight (DW) determination, followed by lipid extraction.

### Expression of CyanMFAT in *Escherichia coli*

The gene WP_015219996 (Cyan10605_2185, 843 bp) was amplified from genomic DNA of Cyan10605 using the oligonucleotides Bn4496 (TTT *GGA TCC* ATG TCA AAT CAA TAT TCA GGC T; italics, restriction site *Bam*HI) and Bn4497 (TTT *CTG CAG* TTA ATC AAT CCC TAG GTA AAG T; italics, *Pst*I) and cloned into pJET1.2 (Thermo Fisher Scientific). The gene was excised with *Bam*HI and *Pst*I, and ligated into pQE-80 L (Qiagen). The construct, which carries an N-terminal His_6_ tag, was transferred to *E. coli* ElectroSHOX (Bioline) cells. Cells were grown in LB medium at 37 °C to an OD_600_ of 0.5–0.6. Protein expression was induced with 0.5 mM isopropyl-β-D-thiogalactopyranoside (IPTG), and the cells were grown at 16 °C with agitation at 180 rpm for 16 h. Protein expression was confirmed by SDS polyacrylamide gel electrophoresis, followed by Coomassie Brilliant Blue staining or Western analysis using HisDetector Nickel-HRP (Kirkegaard & Perry). Bands were visualized by chemiluminescence using the Pierce ECL Plus Western Blotting Substrate kit (Thermo Fisher Scientific).

After induction with IPTG, *E. coli* cells from a 50 mL culture were harvested by centrifugation, suspended in 5 mL of medium and supplemented with 1.5 µmol of dioctanoin (di8:0 diacylglycerol, di8:0-DAG, Sigma-Aldrich/Merck), or phytol (Chemimpex) (substrate dissolved at 0.1% in toluene). The cultures were incubated at 30 °C for 3 h with shaking at 220 rpm. Cells were harvested and washed twice with water. The OD_600_ was determined, followed by lipid extraction from the cell pellets.

For enzyme assays, protein expression was induced as described above. *E. coli* cells were harvested and suspended in homogenization buffer (1 mM EDTA, 200 mM sucrose, 100 mM Tris-HCl, pH 7.4) and homogenized with a sonicator (Bioblock Scientific). The cell extract was centrifuged three times at 10,000 x g for 2 min, and the final supernatant was centrifuged at 28,000 x g for 1 h. The pellet (microsomal membranes) was suspended in homogenization buffer, and the protein concentration was measured using bicinchoninic acid (BCA) assay. The enzymatic assay contained 50 µM palmitoyl-CoA (16:0-CoA), 200 µM dioctanoin or phytol (dissolved in ethanol) and 400 µg of the microsomal fraction in the assay buffer (20 mM MgCl_2_, 0.1% CHAPS, 100 mM Tris-HCl, pH 7.4, 1.25 mg mL^− 1^ BSA, 10 mM Na orthovanadate) in a final volume of 200 µL. After incubation for 20 min at 37˚C, the reaction was terminated, and lipids were extracted by adding 1 mL of methyl tert-butyl ether (MTBE) and 300 µL of methanol.

### Lipid isolation and measurement

Lipids were extracted from cells (Cyan10605, or *E. coli* expressing MFAT) or from acyltransferase assays, with MTBE/methanol/water (10:3:2.5, v/v/v) [22, 24]. Briefly, cells were boiled for 15 min. Internal standards (tri17:0-TAG and tri17:1-TAG, Larodan; 17:0-phytol, synthesized in-house) were added, and the lipids were extracted at room temperature for 1 h. Water was added for phase separation, and the organic phase was harvested after centrifugation. The extraction was repeated and the organic extracts were combined and dried under a nitrogen gas flow. The dried lipids were dissolved in MTBE. Membrane lipids were measured by direct infusion nanospray Q-TOF MS/MS (Agilent Q-TOF 6530) in the positive ionization mode, with chloroform/methanol/300 mM ammonium acetate (300:665:35, v/v/v) as the solvent. For membrane lipid determination, the internal standards were 34:0-MGDG, 36:0-MGDG, 34:0-DGDG, 36:0-DGDG, 34:0-SQDG, 28:0-PG, and 36:0-PG [25]. For fatty acid determination, the total lipids were converted into fatty acid methyl esters (FAME) with methanolic HCl [26]. Thereafter, FAMEs were measured by gas chromatography (GC) with a flame ionization detector (Agilent GC 7890 A) using pentadecanoic acid (15:0) as the internal standard.

Neutral lipids were isolated by solid phase extraction (SPE). The total lipid extract, dissolved in hexane, was loaded onto an SPE column equilibrated with hexane (Macherey & Nagel, 500 mg silica) and subsequently washed with hexane. FAPEs were eluted with hexane/diethyl ether (99:1, v/v) and TAGs with hexane/diethyl ether (95:5, v/v). For acyl-PQH and acyl-PQC isolation, lipids loaded onto a SPE column equilibrated with hexane were directly eluted with hexane/diethyl ether (95:5, v/v). The neutral lipid fractions were dried under nitrogen gas.

TAGs and FAPEs were measured by direct infusion nanospray Q-TOF MS/MS (Agilent Q-TOF 6530) [19, 22] in the positive ionization mode, using chloroform/methanol/300 mM ammonium acetate (300:665:35, v/v/v) as the solvent. Acylated plastoquinols were separated by LC-MS/MS (Agilent Q-TOF 6546) on a Supelcosil ABZ plus column (3 μm, 10 cm x 2.1 mm, Merck) in the positive ionization mode using a gradient of solvent A (tetrahydrofuran/methanol/5 mM ammonium formate, 3:2:5, v/v/v + 0.1% formic acid) and solvent B (tetrahydrofuran/5 mM ammonium formate, 9:1, v/v + 0.1% formic acid), with solvent B increasing from 60% to 100% in 8 min [22]. Due to the lack of commercial standards, acyl-PQH and acyl-PQC levels are presented as normalized signal intensities per mg dry weight.

### Optical density and chlorophyll determination

The optical density (OD_750_) of a 1 mL aliquot of Cyan10605 cells was measured every 24 h at 750 nm using a spectrophotometer (Specord 205, Analytik Jena). Pellets from a 1 mL aliquot of Cyan10605 were extracted with 1 mL of cold methanol at 4˚C, and the mixture was shaken for 20 min. After centrifugation, the supernatant was collected. The absorbance (A) was measured at 665 nm and 720 nm, and the chlorophyll a concentration was calculated based on the following equation:

Chlorophyll a (µg mL^− 1^) = 12.9447 × (A_665_ - A_720_).

### Light and electron microscopy

Transmission electron microscopy was performed at the Microscopy Core Facility of the University of Bonn as previously described [22]. For light microscopy, the cells were harvested by centrifugation and washed with water. Cells were stained with Evans Blue (Merck, Darmstadt) for 1 h at room temperature, as described [27, 28]. Cells were washed and resuspended in water and observed directly with an Olympus BH-2 light microscope.

### Nutrient analysis

Cultures grown under phosphate deprivation or extended cultivation were harvested on day 3, 5, and 7, and on day 14, 28, and 42, respectively. After centrifugation, the supernatant was filtered through a 0.20 μm sterile membrane. The filtrate was acidified with concentrated nitric acid (65%) to a final HNO₃ concentration of 5% (v/v). Samples were subsequently analyzed by inductively coupled plasma optical emission spectrometry (ICP-OES; iCAP Pro X ICP-OES Duo; Thermo Fisher Scientific, Waltham, MA, USA) to determine nutrient concentrations as described [29].

For elemental analysis, aliquots of filtered culture supernatant were transferred into pre-formed tin capsules (two nested 10 × 10 mm capsules per sample). A volume of 550 µL was used to ensure sufficient analyte amounts due to the low nutrient concentrations. Samples were air-dried in a fume hood and subsequently folded into compact pellets. Total nitrogen and carbon contents were determined using a Unicube Elemental Analyzer (Elementar, Langenselbold, Germany). After drying, samples were combusted at 1150 °C under oxygen saturation, and excess oxygen was removed at 850 °C. The resulting gases were separated by gas chromatography and quantified using a thermal conductivity detector [30].

### Statistical analysis

Statistical analysis was performed by Student t- test (unpaired, parametric t test), using GraphPad Prism 9.5.1 (GraphPad Software, Boston, MA, USA). The data are presented as means ± standard deviation of at least three biological replicates (independent cultures growing in separate flasks). A p value of less than 0.05 was considered to be statistically significant.

## Results

### Lipid changes in Cyan10605 during growth at high temperature

**Fig. 1:**
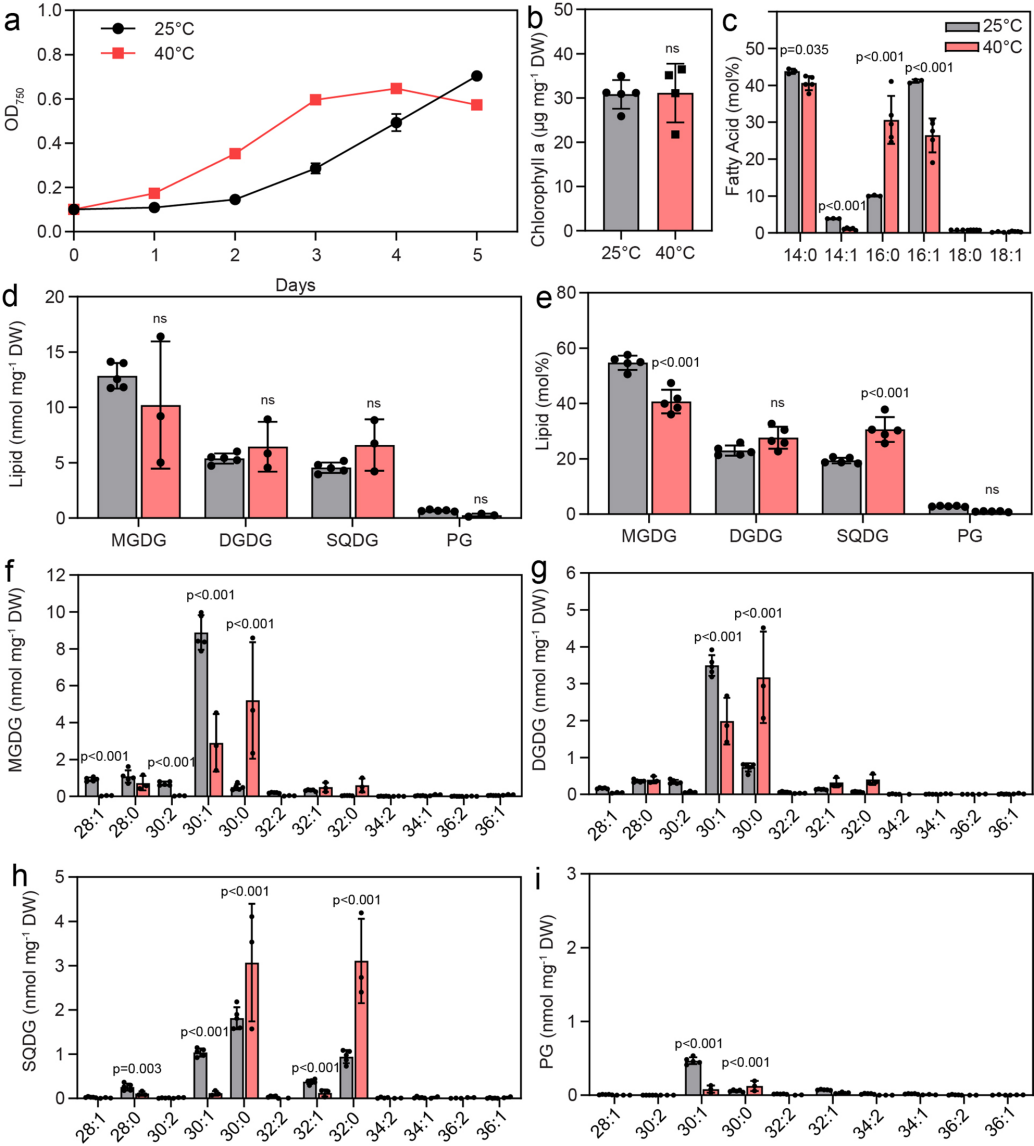
Membrane lipid changes in Cyan10605 during growth at high temperature. Cells of Cyan10605 were grown in shaking flasks at 25 °C and 40 °C for 5 days. (**a**) Growth curves (OD750); (**b**) Chlorophyll a content; (**c**) Fatty acid composition; (**d**, **e**) Membrane lipid composition (nmol mg^− 1^ DW and mol%); (**f**, **g**, **h**, **i**) Molecular species composition of membrane lipids (MGDG, DGDG, SQDG, PG). *N* = 3–5; means ± SD; SD smaller than symbol sizes for some data points; Student’s t-test; p indicated above error bars; ns, not significant; Cyan10605, *Cyanobacterium aponinum* PCC 10605; DGDG, digalactosyldiacylglycerol; DW, dry weight; MGDG, monogalactosyldiacylglycerol; OD_750_, optical density at 750 nm; SQDG, sulfoquinovosyldiacylglycerol; PG, phosphatidylglycerol

The thermophilic strain Cyan10605 was originally isolated from hot springs and can therefore grow at high temperatures of up to 45 °C [5, 7, 15]. The growth of Cyan10605 at 40 °C in BG-11 medium was remarkably accelerated compared to 25 °C until day 4. When the cells grown at 40 °C reached the stationary phase, the cells in the control group (25 °C) were still in the logarithmic phase (Fig. 1a). The chlorophyll a content after growth at 25 °C and 40 °C for 5 days was similar (about 30 µg mg^− 1^ DW) (Fig. 1b).

GC measurements revealed that Cyan10605 contains mostly myristic acid (14:0) and palmitoleic acid (16:1) with minor amounts of myristoleic acid (14:1) and palmitic acid (16:0), but C18 fatty acids (stearic acid, 18:0; oleic acid, 18:1) were largely absent. At 40 °C, the level of 16:0 increased at the expense of the monounsaturated fatty acids 14:1 and 16:1 (Fig. 1c). Membrane lipids were measured by direct infusion mass spectrometry. Like most cyanobacteria, Cyan10605 contains only four membrane lipids, the two galactolipids MGDG and DGDG, the sulfolipid SQDG and the only phospholipid PG (Fig. 1d). The total amount of membrane lipids did not change during growth at 40 °C, but the proportion of SQDG increased, accompanied by a decrease in MGDG (Fig. 1e). The main molecular species of MGDG, DGDG and PG were 30:1 (14:0–16:1, 14:1–16:0) and 30:0 (14:0–16:0). SQDG also contained 30:1 and 30:0 species, in addition to 32:0 (16:0–16:0) and 32:1 (16:0–16:1). In all membrane lipids, a shift from 30:1 species to 30:0 species was observed. Additionally, in SQDG, the amount of 32:0 increased at the expense of 32:1 during growth at 40 °C (Fig. 1f, g, h, i). This result reflects the replacement of monounsaturated acyl groups (14:1, 16:1) with saturated ones (14:0, 16:0) at high temperature.

**Fig. 2:**
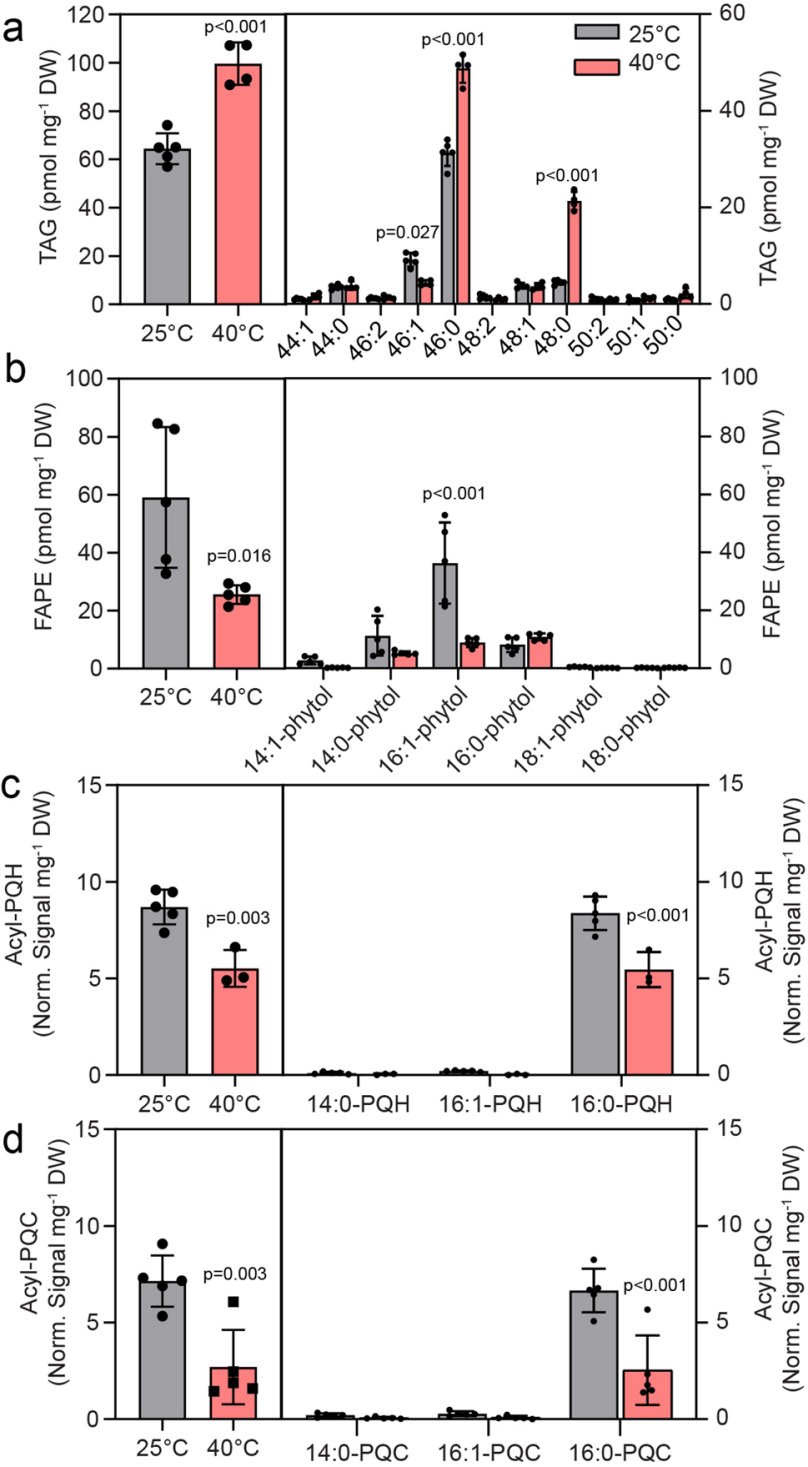
Accumulation of nonpolar lipids in Cyan10605 during growth at high temperature. Cells of Cyan10605 were grown in shaking flasks at 25 °C and 40 °C for 5 days. (**a**) Total TAG content and molecular species composition; (**b**) Total FAPE content and molecular species composition; (**c**) Total acyl-PQH content and molecular species composition; (**d**) Total acyl-PQC content and molecular species composition. *N* = 3–5; means ± SD; Student’s t-test; p indicated above error bars; acyl-PQH, acyl-plastoquinol-9; acyl-PQC, acyl-plastoquinone C; Cyan10605, *Cyanobacterium aponinum* PCC 10605; FAPE, fatty acid phytyl ester; TAG, triacylglycerol

The amounts of TAG and FAPE were around 100-fold lower than those of membrane lipids (Fig. 2a, b). The total TAG content increased by a factor of 1.7 when cells were exposed to high temperature for 5 days. The molecular species composition of TAG in 25 °C-grown cells was dominated by 46:1 (14:0–16:0–16:1, 14:1–16:0–16:0) and 46:0 (14:0–16:0–16:0). At 40 °C, a decrease in 46:1-TAG was accompanied by an increase in 46:0 TAG and 48:0 TAG (16:0–16:0–16:0) (Fig. 2a). The FAPE content of Cyan10605 was reduced by half. The major acyl groups in FAPE of Cyan10605 were 14:0, 16:1 and 16:0, with a specific decrease in 16:1-phytol at 40 °C (Fig. 2b). Cyan10605 also contained acylated forms of plastoquinol, acyl-PQH, carrying the acyl group on C1 or C4 positions of the quinol ring, and acyl-PQC where the acyl group is bonded to a hydroxyl group on the isoprenoid chain of plastoquinone. The two forms of acylated plastoquinols decreased in cells grown at 40 °C compared to 25 °C, and 16:0 was the dominant acyl group in both acyl-PQH and acyl-PQC, with 14:0, 14:1, 16:1 and C18 groups being almost absent (Fig. 2c, d).

### Lipid changes in Cyan10605 during growth in seawater medium

**Fig. 3:**
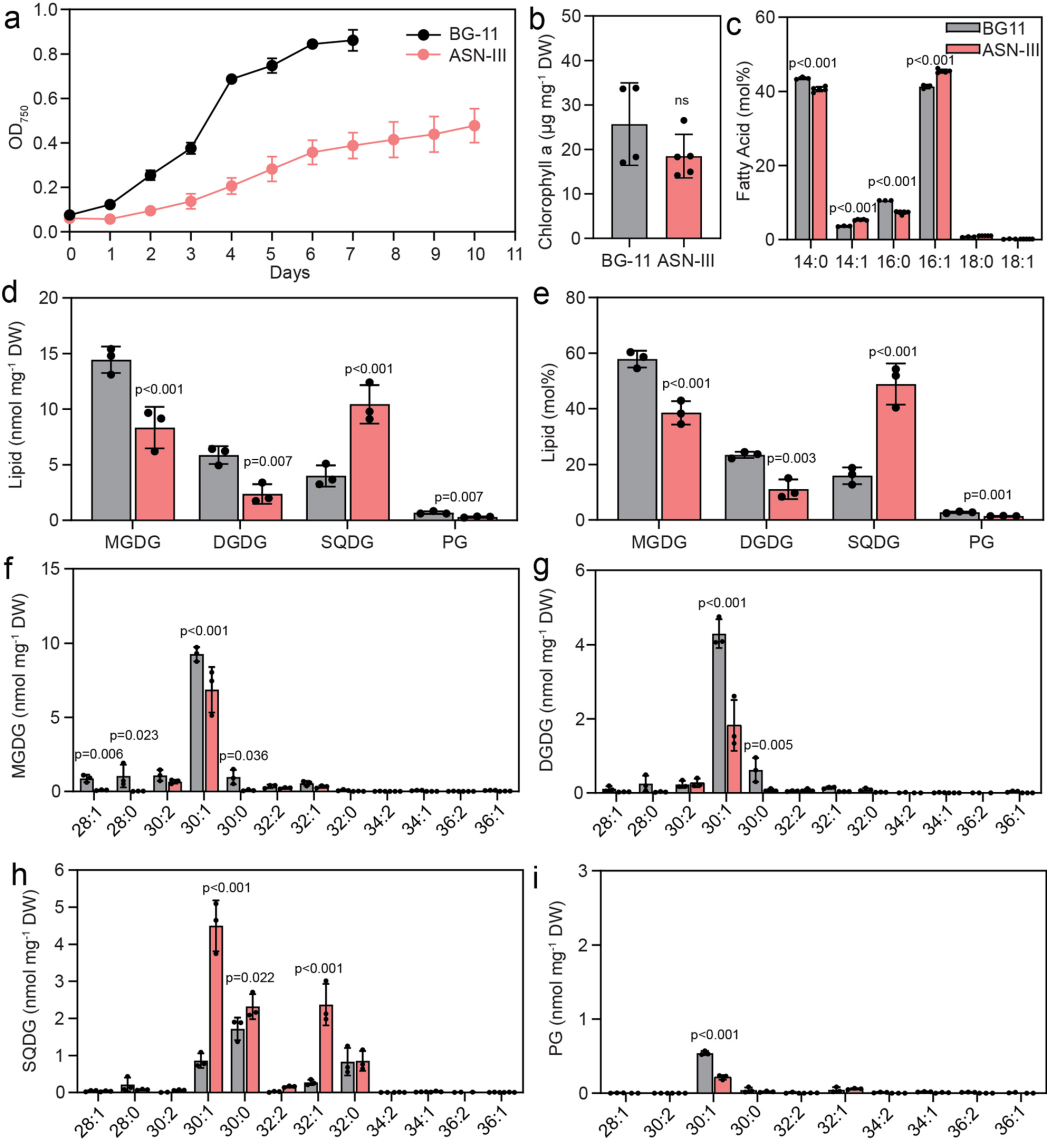
Membrane lipid changes in Cyan10605 during growth in artificial seawater medium. Cells of Cyan10605 were grown in shaking flasks at 25 °C in BG-11 for 7 days or in ASN-III medium for 10 days. (**a**) Growth curves (OD_750_); (**b**) Chlorophyll a content; (**c**) Fatty acid composition; (**d**, **e**) Membrane lipid composition (nmol mg^− 1^ DW and mol%); (**f**, **g**, **h**, **i**) Molecular species composition of membrane lipids (MGDG, DGDG, SQDG, PG). *N* = 3–5; means ± SD; SD smaller than symbol sizes for some data points; Student’s t-test; p indicated above error bars; Cyan10605, *Cyanobacterium aponinum* PCC 10605; DGDG, digalactosyldiacylglycerol; DW, dry weight; MGDG, monogalactosyldiacylglycerol; OD_750_, optical density at 750 nm; PG, phosphatidylglycerol; SQDG, sulfoquinovosyldiacylglycerol

**Fig. 4:**
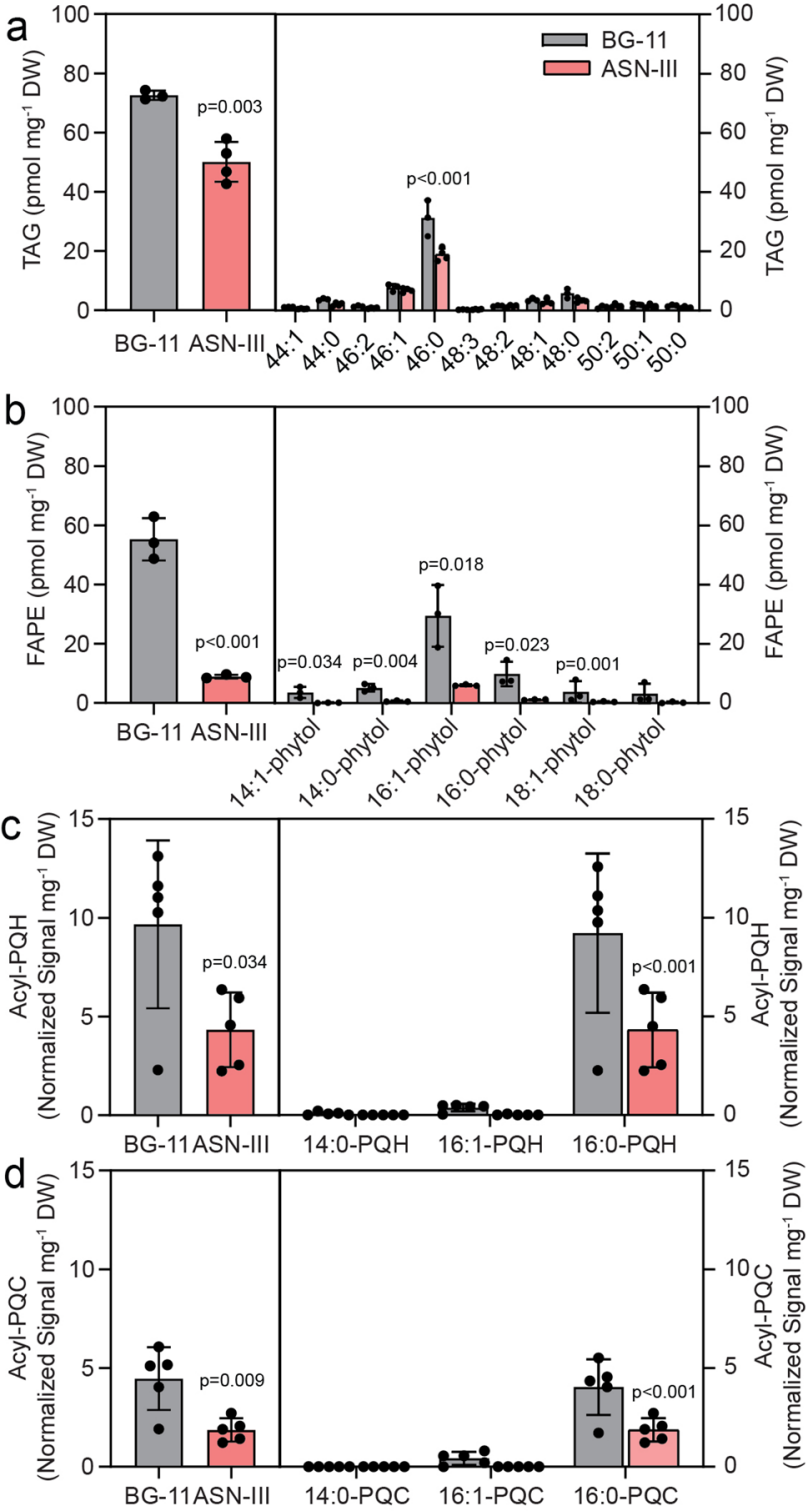
Accumulation of nonpolar lipids in Cyan10605 during growth in artificial seawater medium. Cells of Cyan10605 were grown in shaking flasks at 25 °C in BG-11 medium for 7 days or ASN-III medium for 10 days. (**a**) Total TAG and molecular species composition; (**b**) Total FAPE content and molecular species composition; (**c**) Total acyl-PQH content and molecular species composition; (**d**) Total acyl-PQC content and molecular species composition. *N* = 3–5; means ± SD; SD smaller than symbol sizes for some data points; Student’s t-test; p indicated above error bars; acyl-PQH, acyl-plastoquinol-9; acyl-PQC, acyl-plastoquinone C; Cyan10605, *Cyanobacterium aponinum* PCC 10605; DW, dry weight; FAPE, fatty acid phytyl ester; TAG, triacylglycerol

Cyan10605 is a halotolerant strain and can therefore grow in freshwater and seawater setups. To study the lipid responses of the cells to high salt environments, the cells were grown in artificial seawater (ASN-III) and control (BG-11) media at 25 °C. The cells grew faster in BG-11 medium and reached an OD_750_ of 0.8 after 5 days. The growth of Cyan10605 was retarded in ASN-III medium, as cells reached an OD_750_ of only 0.5 after 10 days (Fig. 3a). The chlorophyll a content on the final day of cultivation was not significantly different (Fig. 3b). The fatty acid composition displayed a shift towards monounsaturated fatty acids (14:1, 16:1) at the expense of saturated fatty acids (14:0, 16:0) in ASN-III medium (Fig. 3c). The amounts of the two galactolipids, MGDG and DGDG, were drastically decreased, while the PG content remained low, and the amount of SQDG was strongly increased in ASN-III medium (Fig. 3d, e). While there was a decline of 30:1 and 30:0 molecular species of MGDG, DGDG and PG, these two species as well as 32:1 were strongly increased in SQDG, consistent with the shift towards 14:1 and 16:1 in the total fatty acid composition (Fig. 3). The amounts of TAG and FAPE, in particular the main molecular species 46:0 TAG and 16:1-phytol, were reduced during growth in ASN-III medium. Furthermore, decreased levels of acyl-PQH and acyl-PQC were observed (Fig. 4). Taken together, the cells displayed a retarded growth in artificial seawater accompanied by a decrease in galactolipids, an increase in monounsaturated fatty acid-rich SQDG and a decrease in nonpolar lipid contents.

### Membrane lipid composition in Cyan10605 during phosphate limitation

**Fig. 5:**
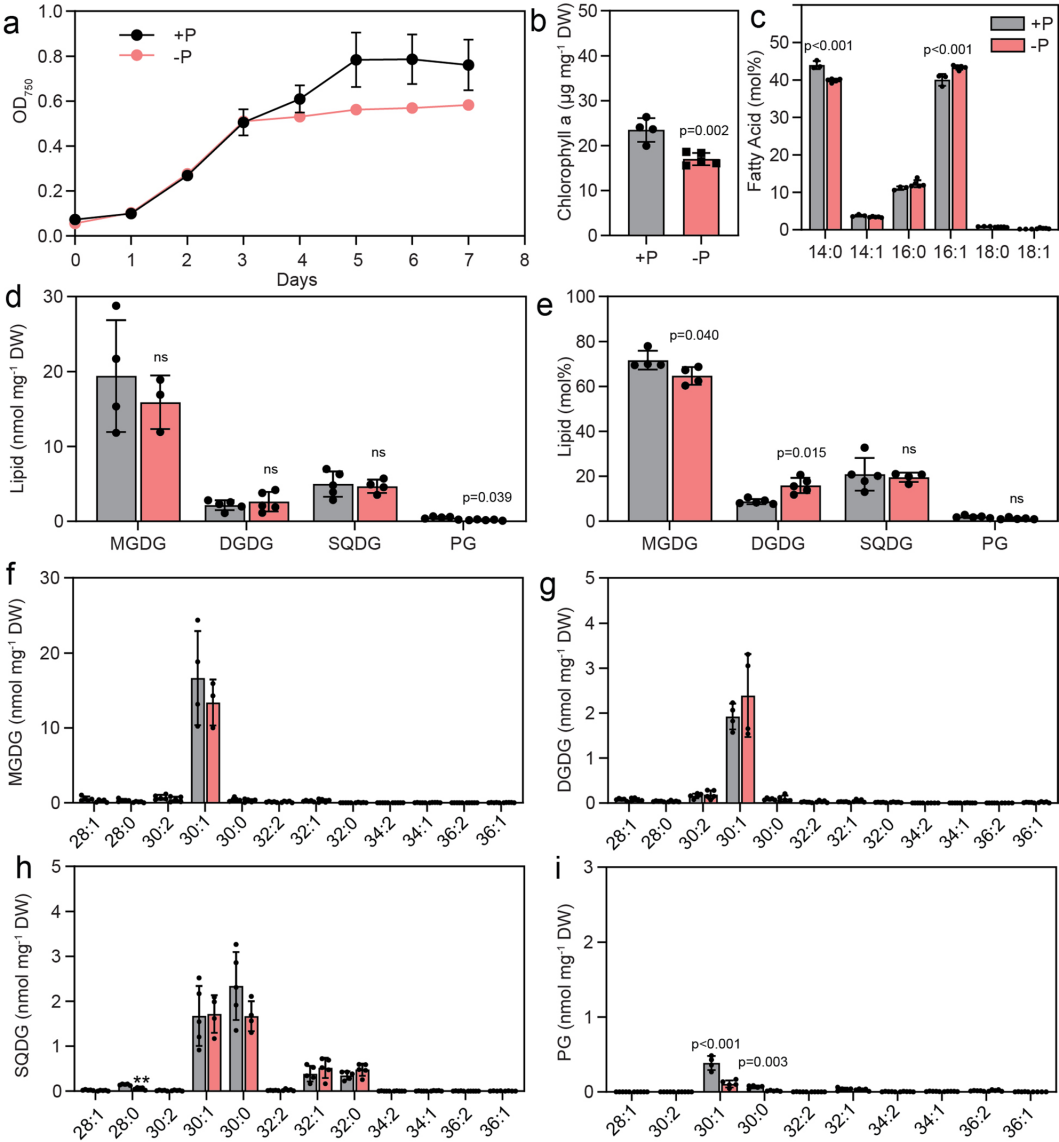
Membrane lipid changes in Cyan10605 during growth under phosphate deprivation. Cells of Cyan10605 were grown in shaking flasks at 25 °C in the presence (+ P) or absence (-P) of phosphate for 7 days. (**a**) Growth curves (OD_750_); (**b**) Chlorophyll a content; (**c**) Fatty acid composition of the cells; (**d**, **e**) Membrane lipid composition (nmol mg^− 1^ DW and mol%); (**f**, **g**, **h**, **i**) Molecular species composition of membrane lipids (MGDG, DGDG, SQDG, PG). *N* = 3–5; means ± SD; SD smaller than symbol sizes for some data points; Student’s t-test; p indicated above error bars; ns, not significant; Cyan10605, *Cyanobacterium aponinum* PCC 10605; DGDG, digalactosyldiacylglycerol; DW, dry weight; MGDG, monogalactosyldiacylglycerol; OD_750_, optical density at 750 nm; PG, phosphatidylglycerol; SQDG, sulfoquinovosyldiacylglycerol

**Fig. 6:**
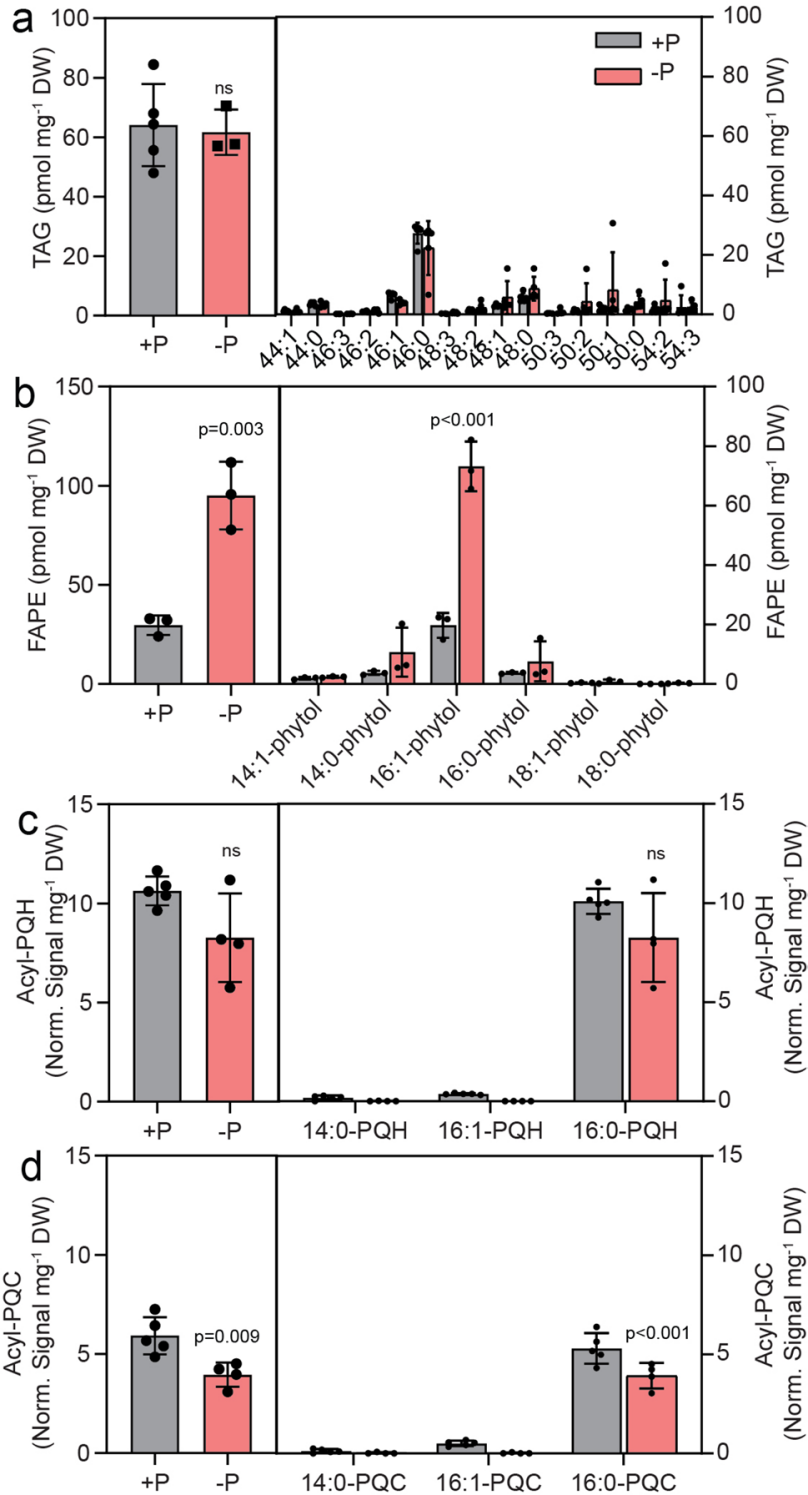
Accumulation of nonpolar lipids in Cyan10605 during growth under phosphate deprivation. Cells of Cyan10605 were grown in shaking flasks at 25 °C in the presence (+ P) or absence (-P) of phosphate for 7 days. (**a**) Total TAG content and molecular species composition; (**b**) Total FAPE content and molecular species composition; (**c**) Total acyl-PQH content and molecular species composition; (**d**) Total acyl-PQC content and molecular species composition. *N* = 3–5; means ± SD; Student’s t-test; p indicated above error bars; ns, not significant; acyl-PQH, acyl-plastoquinol-9; acyl-PQC, acyl-plastoquinone C; Cyan10605, *Cyanobacterium aponinum* PCC 10605; DW, dry weight; FAPE, fatty acid phytyl ester; TAG, triacylglycerol

In plants and many cyanobacteria, growth under phosphate deprivation causes a decrease in phospholipids, including PG, and a corresponding increase in DGDG and SQDG, which replace the phospholipids [31, 32]. Growth of Cyan10605 cells in medium with or without phosphate was similar until day 3, when the phosphate-deprived cells reached stationary phase at OD_750_ ~0.5, while the phosphate-replete cells continued to grow until they reached the stationary phase on day 5 (OD_750_ ~0.8) (Fig. 5a). The chlorophyll a content of phosphate-deprived cells was reduced to ~ 17 µg mg^− 1^ DW (Fig. 5b). Phosphate deprivation caused an increase in the proportion of 16:1 at the expense of the 14:0 fatty acid (Fig. 5c). The relative membrane lipid composition of the cells remained similar during phosphate deprivation except for a slight increase in DGDG and a decrease in MGDG (Fig. 5e). The PG content was very low and did not significantly change (Fig. 5d, e). Therefore, in contrast to other cyanobacteria and plants, phosphate deprivation has only a minor impact on the membrane lipid composition of Cyan10605. The amounts of TAG in Cyan10605 did not change during 7 days of growth under phosphate deprivation (Fig. 6a). However, we found an accumulation of total FAPE content (by 2.2-fold), with 16:1-phytol being the predominant molecular species (Fig. 6b). Furthermore, the content of acyl-PQH remained unchanged, while the content of acyl-PQC declined under phosphate limitation (Fig. 6c, d).

### The effect of nitrogen deprivation on lipid composition in Cyan10605

**Fig. 7:**
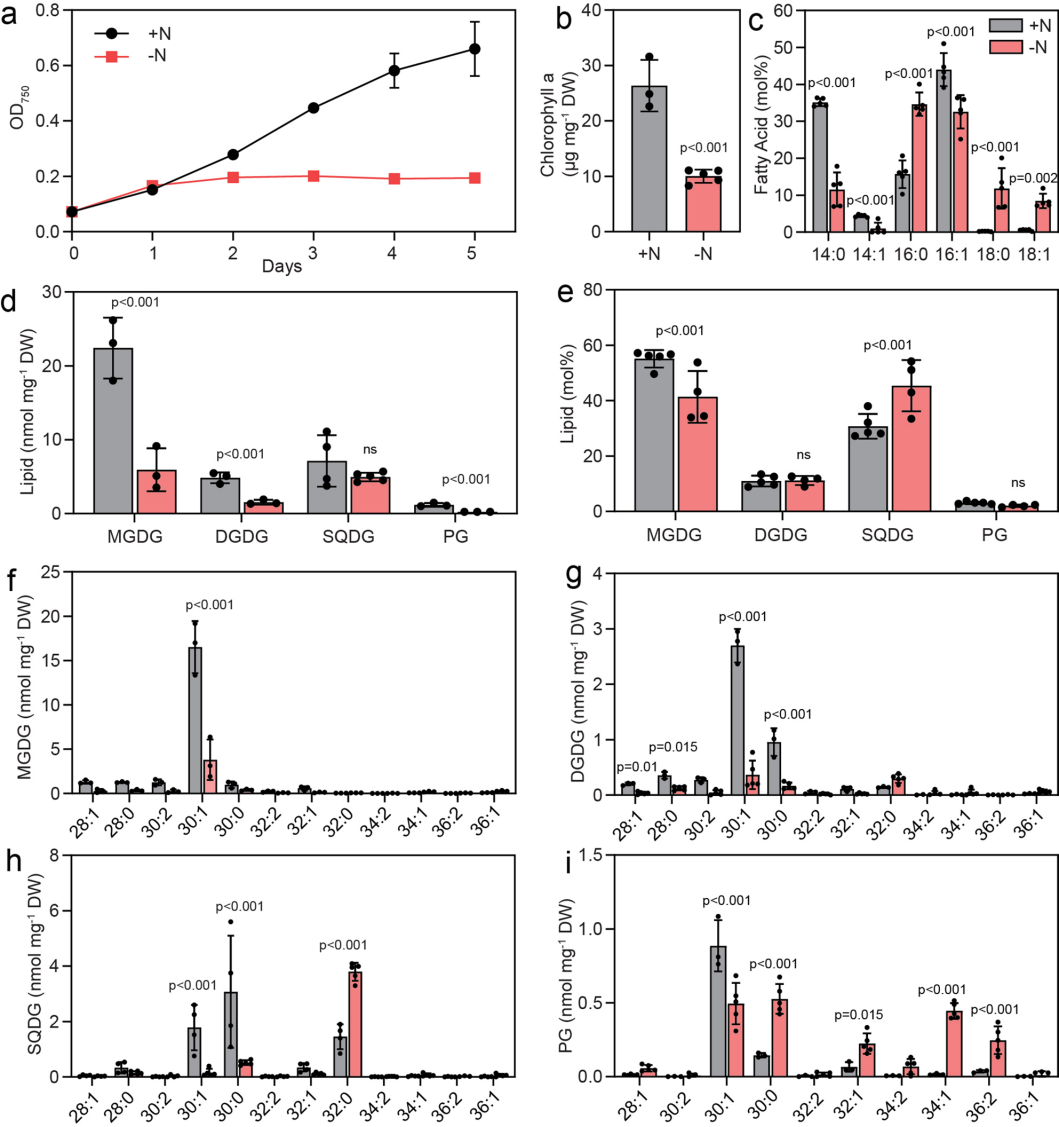
Membrane lipid changes in Cyan10605 during growth during nitrogen deprivation. Cells of Cyan10605 were grown in shaking flasks at 25 °C in the presence (+ N) or absence (-N) of nitrogen for 5 days. (**a**) Growth curves (OD_750_); (**b**) Chlorophyll a content; (**c**) Fatty acid composition of the cells; (**d**, **e**) Membrane lipid composition (nmol mg^− 1^ DW and mol%) ; (**f**, **g**, **h**, **i**) Molecular species composition of membrane lipids (MGDG, DGDG, SQDG, PG). *N* = 3–5; means ± SD; ns, not significant; SD smaller than symbol sizes for some data points; Student’s t-test; *p* indicated above error bars; Cyan10605, *Cyanobacterium aponinum* PCC 10605; DGDG, digalactosyldiacylglycerol; DW, dry weight; MGDG, monogalactosyldiacylglycerol; OD_750_, optical density at 750 nm; PG, phosphatidylglycerol; SQDG, sulfoquinovosyldiacylglycerol

**Fig. 8:**
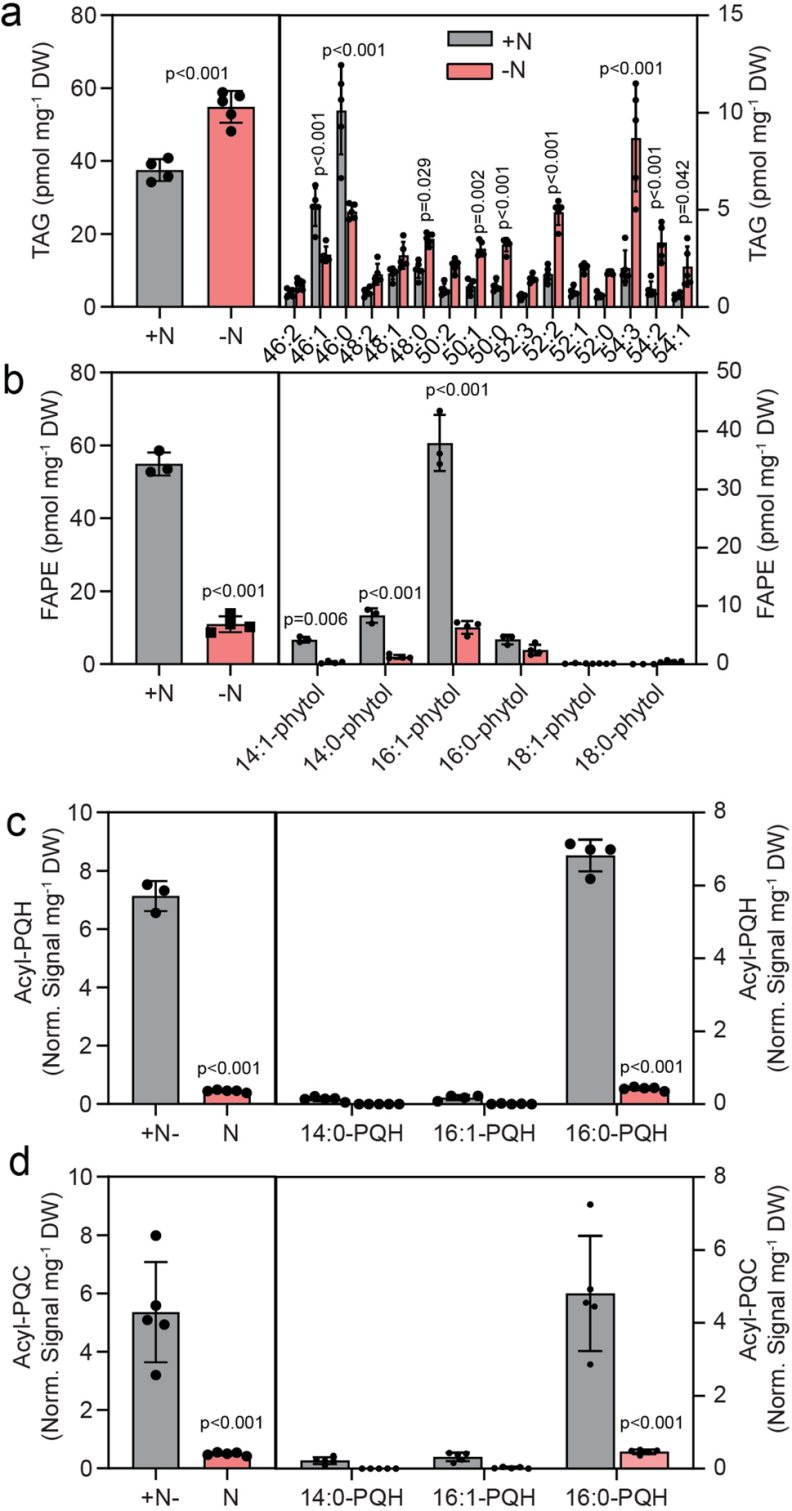
Accumulation of nonpolar lipids in Cyan10605 during growth under nitrogen deprivation. Cells of Cyan10605 were grown in shaking flasks at 25 °C in the presence (+ N) or absence (-N) of nitrogen for 5 days. (**a**) Total TAG content and molecular species composition; (**b**) Total FAPE content and molecular species composition; (**c**) Total acyl-PQH content and molecular species composition; (**d**) Total acyl-PQC content and molecular species composition. *N* = 3–5; means ± SD; Student’s t-test; p indicated above error bars; acyl-PQH, acyl-plastoquinol-9; acyl-PQC, acyl-plastoquinone C; Cyan10605, *Cyanobacterium aponinum* PCC 10605; DW, dry weight; FAPE, fatty acid phytyl ester; TAG, triacylglycerol

Nitrogen deprivation is known to have a strong impact on cyanobacterial metabolism. Growth of Cyan10605 cells was strongly suppressed under –N conditions, as growth ceased after 2 days at OD_750_ of ~ 0.2, while cells reached stationary phase at OD_750_ ~0.6 after five days under + N conditions (Fig. 7a). Cells grown under + N were dark green, while –N cells were pale green, in agreement with the strong decrease in chlorophyll a content under nitrogen starvation (Fig. 7b). GC measurements revealed that under -N conditions, the amounts of 16:0, 18:0 and 18:1 were increased in Cyan10605, while the contents of 14:0, 14:1 and 16:1 were decreased, indicating a shift from medium chain (C14) to long chain (C18) fatty acids (Fig. 7c). During N deprivation, the absolute amounts of MGDG, DGDG and PG were strongly decreased but the SQDG content remained unchanged, indicating that the photosynthetic membranes were to a large extent degraded (Fig. 7d). The relative composition of membrane lipids was altered, with an elevation of SQDG at the expense of MGDG (Fig. 7e). In the two galactolipids, the amounts of the main molecular species (30:1 MGDG, 30:1 DGDG, 30:0 DGDG) were decreased. Similarly, the contents of 30:1 SQDG and 30:0 SQDG were decreased, but 32:0 SQDG was strongly increased. PG displayed a shift from 30:1 PG to 30:0 PG, 32:1 PG, 34:1 PG and 36:2 PG species. The changes in molecular species composition are in line with a shift from C14 containing molecular species (30:1, 30:0) to C16 and C18 containing species (32:0, 32:1, 34:1, 36:2) (Fig. 7c-i). Under N deprivation, the amount of TAG was increased by ~ 46%. The molecular species composition of TAG was shifted to long chain molecules with decreased amounts of 46:1 and 46:0 (C14, C16 acyl group-containing TAG species) and increased amounts of 50:1, 50:0, 52:2 (C16-, C18-containing TAG species), and 54:3, 54:2, 54:1 (C18-containing TAG species) (Fig. 8a), in agreement with the shift from C14 to C18 acyl groups (Fig. 7c). The amounts of FAPE, including all molecular species, were strongly decreased (Fig. 8b). Both acyl-PQH and acyl-PQC contents were drastically decreased to ~ 0.5 normalized signal mg^− 1^ DW (Fig. 8c and d).

### Accumulation of TAG and FAPE during extended growth in Cyan10605

**Fig. 9:**
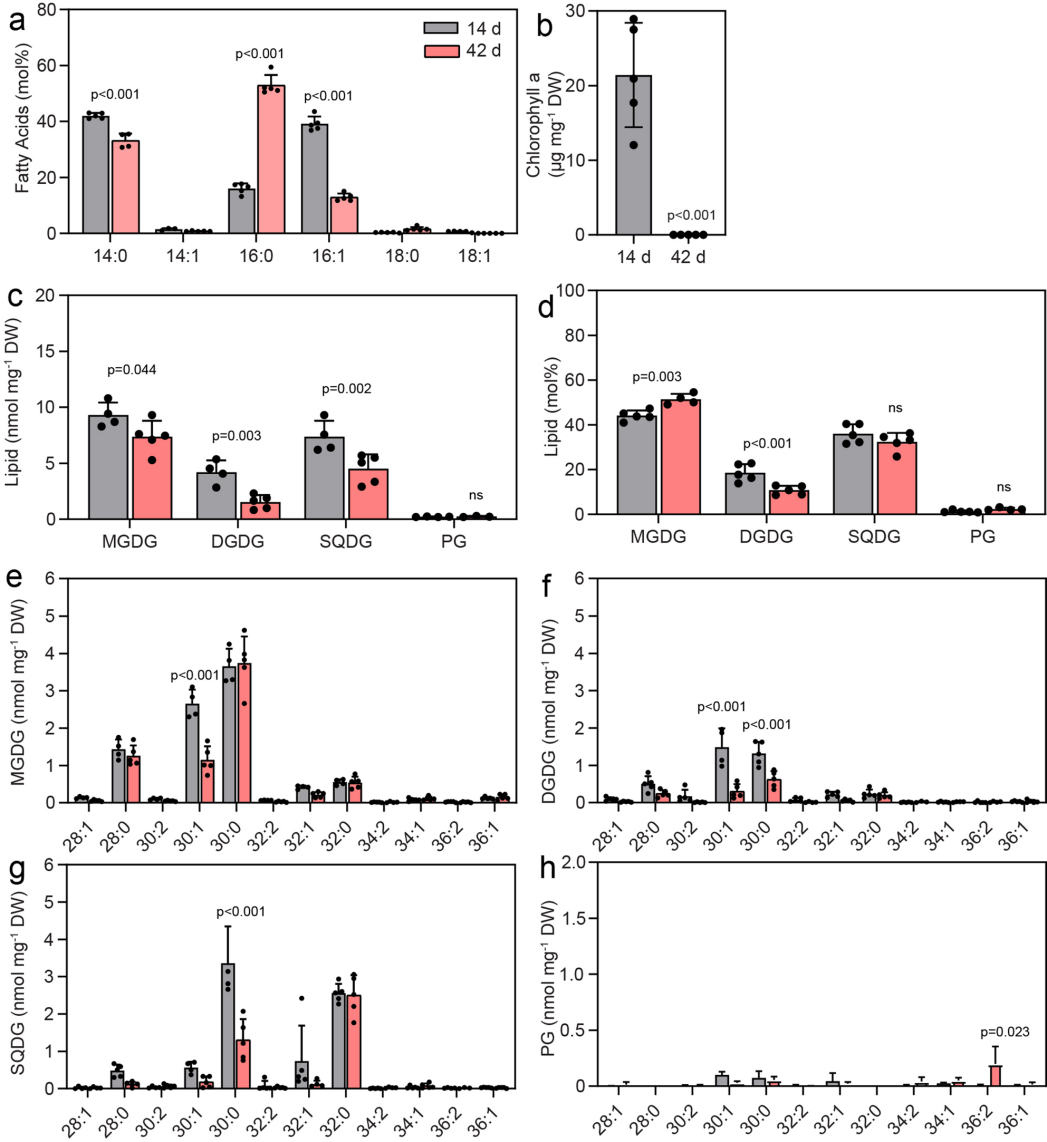
Membrane lipid changes in Cyan10605 after extended cultivation. Cells of Cyan10605 were grown without shaking at 25 °C for 14 days or 42 days. (**a**) Chlorophyll a content; (**b**) Fatty acid composition; (**c**, **d**) Membrane lipid composition (nmol mg^− 1^ DW and mol%); (**e**, **f**, **g**, **h**) Molecular species composition of membrane lipids (MGDG, DGDG, SQDG, PG). *N* = 5; means ± SD; Student’s t-test; p indicated above error bars; Cyan10605, *Cyanobacterium aponinum* PCC 10605; DW, dry weight; DGDG, digalactosyldiacylglycerol; MGDG, monogalactosyldiacylglycerol; OD_750_, optical density at 750 nm; PG, phosphatidylglycerol; SQDG, sulfoquinovosyldiacylglycerol

**Fig. 10:**
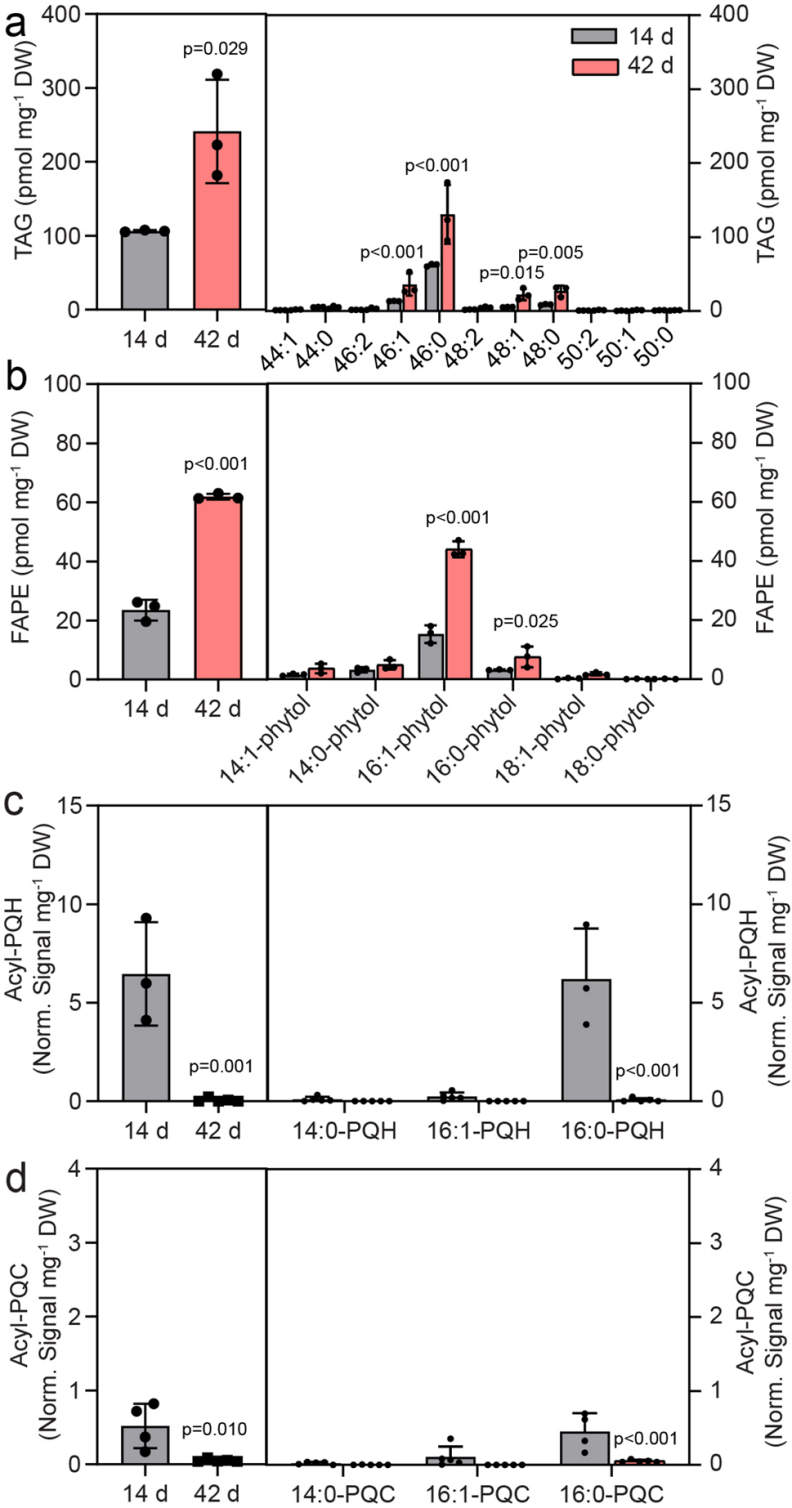
Accumulation of non-polar lipids in Cyan10605 after extended cultivation. Cells of Cyan10605 were grown without shaking at 25 °C for 14 days or 42 days. (**a**) Total TAG content and molecular species composition; (**b**) Total FAPE content and molecular species composition; (**c**) Total acyl-PQH content and molecular species composition; (**d**) Total acyl-PQC content and molecular species composition. *N* = 3–5; means ± SD; Student’s t-test; p indicated above error bars; acyl-PQH, acyl-plastoquinol-9; acyl-PQC, acyl-plastoquinone; Cyan10605, *Cyanobacterium aponinum* PCC 10605; DW, dry weight; FAPE, fatty acid phytyl ester; C TAG, triacylglycerol

To determine whether extended cultivation results in changes in the composition of membrane lipids and nonpolar lipids, Cyan10605 cells were grown at 25 °C for 14 or 42 days in modified BG-11 medium as static cultures. After a long growth period of 42 days, the chlorophyll a content was strongly reduced to negligible levels (Fig. 9a). Analysis of fatty acids demonstrated the accumulation of 16:0 (by 2.3-fold) at the expense of 14:0 and 16:1 (Fig. 9b). After 14 days of growth without agitation, MGDG and SQDG were the predominant lipid classes, followed by DGDG, with PG only accounting for 1.3 mol% (Fig. 9c, d), consistent with the low PG proportion in Cyan10605 under other growth conditions (Figs. 1, 3, 5 and 7). After 42 days, a strong decrease in the contents of galactolipids (MGDG and DGDG) and the sulfolipid (SQDG) was observed, with the total amount of membrane lipids declining from 21 nmol mg^− 1^ DW on day 14 to 12 nmol mg^− 1^ DW on day 42 (Fig. 9c). Moreover, the molecular species 30:1 of MGDG and DGDG, and 30:0 of DGDG and SQDG were decreased, while 32:0 of the three lipids remained unaffected (Fig. 9e-h). A remarkable accumulation of TAG and FAPE reaching 242 pmol mg^− 1^ DW and 62 pmol mg^− 1^ DW, respectively, was observed after 42 d of cultivation. Moreover, 46:0, 46:1, 48:0, and 48:1 were the most abundant molecular species of TAG, and 16:1 and 16:0 were the most abundant species of FAPE (Fig. 10a, b). Concomitantly, plastoquinol ester levels were strongly decreased, with acyl-PQH and acyl-PQC amounts dropping to almost undetectable levels of 0.07 and 0.06 (normalized signal mg^− 1^ DW), respectively (Fig. 10c, d).

### Ultrastructure of Cyan10605 cells under different growth conditions

**Fig. 11:**
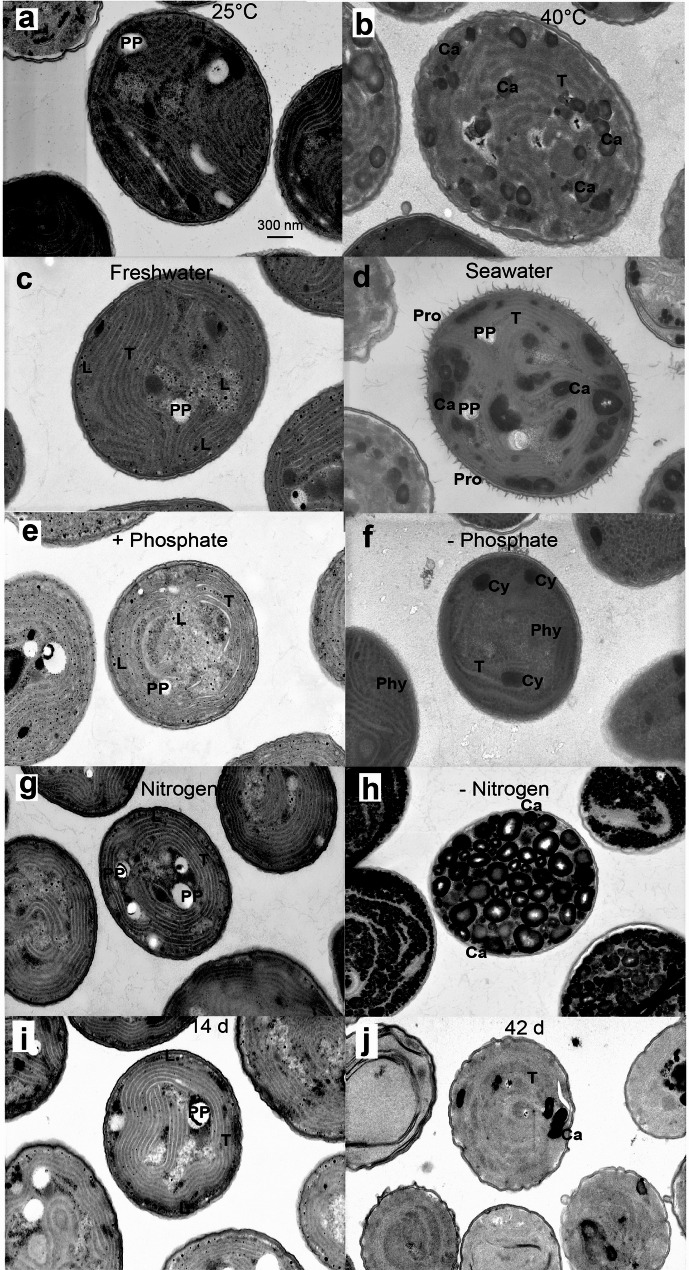
Ultrastructure of Cyan10605 cells grown under different conditionss. (**a**, **b**) Cells grown on BG-11 medium at 25–40 °C; (**c**, **d**) Cells grown on BG-11 or ASN-III medium; (**e**, **f**) Cells grown in the presence or absence of phosphate. (**g**, **h**) Cells grown in the presence or absence of nitrogen. (**i**, **j**) Cells grown without shaking at 25 °C for 14 days or 42 days. Ca, carboxysome; Cy, cyanophycin granule; L, lipid droplet; Phy, phycobilisomes; PP, polyphosphate; Pro, protrusions; T, thylakoid

The ultrastructure of Cyan10605 cells was studied by transmission electron microscopy. Cells grown under control conditions (modified BG-11, 25 °C) exhibited well-organized thylakoid membranes associated with phycobilisomes, lipid droplets distributed within the cytosol, and non-osmophilic polyphosphate bodies (Fig. 11a, c, e, g, i). At an elevated temperature of 40 °C, lipid droplets disappeared, carboxysomes increased in number and the thylakoid membranes were rearranged (Fig. 11b). Similarly, in cells grown in seawater medium, lipid droplets were reduced and carboxysomes accumulated. Furthermore, fimbriae-like protrusions on the outer membrane became visible (Fig. 11d). Phosphate deprivation caused the loss of polyphosphate bodies. Cyanophycin granules were found in the majority of cells, and phycobilisomes became more apparent (Fig. 11f). Under nitrogen starvation, the thylakoid membranes were mostly degraded, in agreement with the decreased chlorophyll content (Fig. 7b). Furthermore, numerous carboxysomes were observed in the cytosol (Fig. 11h). Extended growth led to an increased presence of carboxysomes and degradation of thylakoid membranes, as shown by a decrease in the number and packing density of thylakoids (Fig. 11j). This observation is consistent with the strong decrease in total membrane lipid content after 42 days of growth (Fig. 9b, c).

### Nonpolar lipid synthesis by the acyltransferase MFAT in Cyan10605

**Fig. 12:**
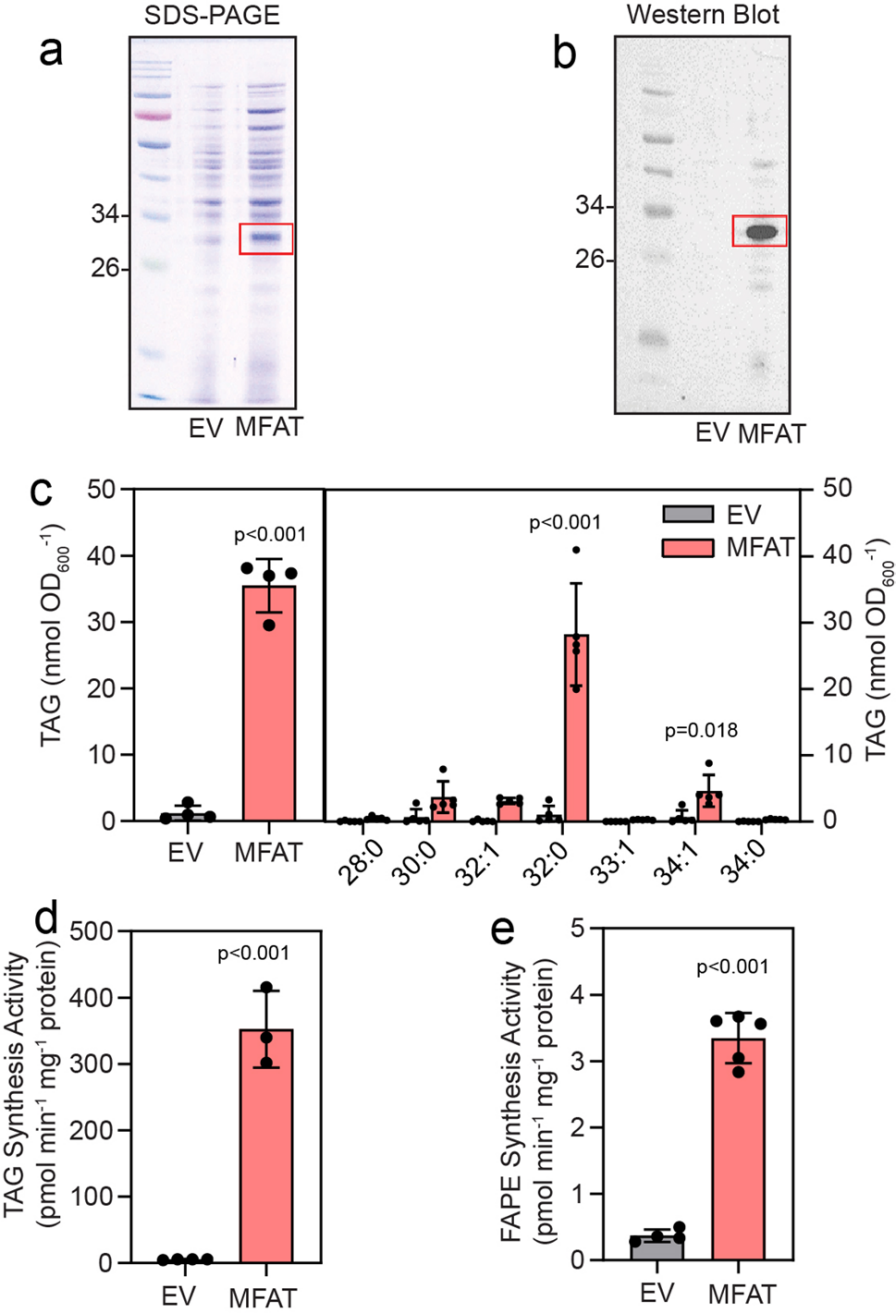
Triacylglycerol and fatty acid phytyl ester synthesis activity of the acyltransferase MFAT from Cyan10605. (**a**) MFAT from Cyan10605 was expressed in *E. coli*, proteins separated by SDS polyacrylamide gel electrophoresis and stained with Coomassie Brilliant blue. (**b**) MFAT expressed in *E. coli* was detected by Western analysis using the Ni-horseradish peroxidase conjugate. The calculated mass of the MFAT polypeptide (including His_6_-tag) is 32.04 kDa. (**c**) MFAT from Cyan10605 was expressed in *E. coli*, the cells supplemented with dioctanoin (di-8:0 DAG), and total TAG content and molecular species composition were determined. (**d**) In vitro acyltransferase activity of MFAT after expression in *E. coli* with dioctanoin and palmitoyl-CoA. (**e**) In vitro acyltransferase activity of MFAT after expression in *E. coli* with phytol and palmitoyl-CoA. TAG and FAPE were measured by direct infusion mass spectrometry. *N* = 3–5; means ± SD; Student’s t-test; p indicated above error bars; EV, empty vector; FAPE, fatty acid phytyl ester; MFAT, multifunctional acyltransferase; SDS-PAGE, sodium dodecyl sulfate polyacrylamide gel electrophoresis; TAG, triacylglycerol

The MFAT proteins Slr2103 from *Synechocystis* sp. PCC 6803 and A0918 from *Synechococcus* sp. PCC 7002 are involved in the synthesis of TAG, FAPE and acylated plastoquinols, as shown by analysis of the cyanobacterial mutants and expression in *E. coli* [19, 22]. The genome of Cyan10605 contains one MFAT-like gene (WP_015219996; Cyan10605_2185) which shows 55.6% identity at the amino acid level with the Slr2103 sequence. The gene Cyan10605_2185 was expressed in *E. coli* (Fig. 12a, b; Fig. S3). The cells were supplemented with dioctanoin, followed by lipid isolation and TAG measurements by mass spectrometry. The MFAT protein catalyzed TAG synthesis, incorporating mostly 16:0 and 18:1 fatty acids from *E. coli* into TAG (Fig. 12c). Next, total protein was isolated from *E. coli* cells expressing MFAT and used for acyltransferase assays with dioctanoin and palmitoyl-CoA (16:0-CoA). The heterologous MFAT protein showed high TAG synthesis activity (352 pmol min^− 1^ mg^− 1^ protein) (Fig. 12d). Furthermore, acyltransferase assays with phytol and 16:0-CoA revealed that the MFAT exhibited FAPE synthesis activity of 3.37 pmol min^− 1^ mg^− 1^ protein (Fig. 12e). Attempts to demonstrate plastoquinol ester synthesis activity of the MFAT by supplementing decylplastoquinone, a plastoquinone analog, to the cells, or by enzymatic assay with the reduced form of decylplastoquinol, were not successful. Taken together, the MFAT from Cyan10605 reveals TAG and FAPE synthesis activity, with substantially higher activity toward dioctanoin than toward phytol.

## Discussion

*C. aponinum* is a biotechnologically important cyanobacterium based on its flexibility to grow under extreme conditions including high temperature and salinity, and because of its capacity to produce value-added compounds such as phycocyanin and exopolysaccharides. We show here that *C. aponinum* has evolved characteristic lipid responses, including changes in membrane lipid and nonpolar lipid contents as well as in the degree of acyl unsaturation and chain length, that likely enable the cells to grow under such extreme conditions. These results are crucial to optimize the application of Cyan10605 for the production of value-added compounds. Moreover, we investigated its growth, morphology, and lipidomic changes in response to different growth conditions, which provide insights into the adaptive mechanism to extreme conditions and will be important to establish Cyan10605 culture conditions for outdoor or mass cultivation [8].

### Fatty acids in Cyan10605

While no lipid data for Cyan10605 were available, different results were previously obtained for the acyl composition of other *C. aponinum* strains. Lipids of *C. aponinum* (Ankara strain) were rich in saturated (18.3% of 10:0; 8.3% of 14:0; 21.9% of 16:0; 29.4% of 18:0) and monounsaturated fatty acids (6.6% of 16:1; 2.9% of 17:1; 5.0% of 18:1) [8]. Another *C. aponinum* strain (UTEX 3222) contained 39.2% of 14:0, 17.1% of 16:0, 30.7% of 16:1, and minor amounts of C18 fatty acids [9]. *Cyanobacterium* sp. IPPAS B-1200 contained 30.0% of 14:0, 17.0% of 16:0, 10.0% of 14:1 and 40.0% of 16:1 [10, 33]. Figure 1c shows that Cyan10605 contains mostly 14:0, 14:1, 16:0 and 16:1, but lacks C17 or C18 fatty acids, similar to strains UTEX 3222 and IPPAS B-1200, but different from the Ankara strain. The high contents of C14 fatty acids in *Cyanobacterium* sp. IPPAS B-1200 were explained by the specificities of lysophosphatidic acid acyltransferase (LPAAT) and Δ9-fatty acid desaturase (Δ9-FAD) [10]. LPAAT in strain IPPAS B-1200 showed a unique preference for 14:0, during the acylation of lysophosphatidic acid to yield phosphatidic acid, a precursor for glycerolipid synthesis. However, in most cyanobacteria, lysophosphatidic acid is esterified exclusively with a C16 fatty acid. The Δ9-FAD of strain IPPAS B-1200 displayed a broad chain length specificity for C14 and C16 saturated fatty acids [34, 35]. The absence of acyl groups longer than 16 carbon atoms from Cyan10605 under most growth conditions, except nitrogen deprivation, presumably results in thinner thylakoid and plasma membranes, which might enhance stability and support protein functions under stress conditions. Decreasing acyl‑chain length reduces bilayer thickness and alters membrane packing, to adjust membrane thickness to transmembrane domains of embedded proteins and thereby support protein function [34–36]. Membranes containing mainly C16 and C18 acyl groups seem to be specific for marine cyanobacteria as shown for *Synechococcus* sp. WH7803 [37]. Under low temperatures, this strain contains even shorter acyl chains, which results in membrane thinning and altered desaturation levels, collectively regulating membrane fluidity.

### Lipid responses in Cyan10605 to high temperature

In agreement with the capacity of Cyan10605 for thermophilic cultivation, the cells showed improved growth at 40 °C compared with 25 °C (Fig. 1a). At 40 °C, thylakoid membranes were rearranged, lipid droplets disappeared, and carboxysomes accumulated (Fig. 11a). Carboxysomes are bacterial micro-compartments containing ribulose-1,5-bisphosphate carboxylase/oxygenase (RuBisCO) and carbonic anhydrase. Carboxysomes concentrate CO_2_ around RuBisCO to suppress photooxidation under low CO_2_/high O_2_ concentrations. The increased requirement for carboxysomes presumably stems from the fact that the solubility ratio of O_2_/CO_2_ increases with temperature, because O_2_ is more soluble in water than CO_2_ at high temperatures [38]. While the total amount of membrane lipids in Cyan10605 did not change, a shift from unsaturated (16:1) to saturated (16:0) acyl groups and a decline in the ratio of MGDG to SQDG were observed during growth at high temperatures (Fig. 1c-e). In the thermophilic cyanobacterium *Mastigocladus laminosu*s, the proportions of MGDG, SQDG and PG were decreased at 55 °C, while the proportion of DGDG was increased [16]. Similar to Cyan10605, the proportions of monounsaturated fatty acids (16:1, 18:1) were decreased, and saturated fatty acids (16:0, 18:0) accumulated at high temperatures in *M. laminosus*, *Synechococcus vulcanus* and *Synechococcus elongatus* (B-267). Notably in *S. vulcanus*, the major changes in fatty acid unsaturation were observed in SQDG, rather than in MGDG and DGDG [16–18]. In addition, the amount of DGDG was elevated at the expense of MGDG at 55 °C compared to lower temperatures in *S. vulcanus* [17]. The importance of SQDG is supported by the finding that disruption of the *sqdB* gene involved in SQDG synthesis in *Thermosynechococcus elongatus* BP-1, affects the formation of photosystem (PS) I trimers and PSII dimers, and the energy transfer in phycobilisomes [39, 40]. Therefore, a common response of thermophilic cyanobacteria is the replacement of (mono-)unsaturated with saturated acyl groups, because saturated acyl groups counteract the increased membrane fluidity at high temperatures [34, 35, 41]. Furthermore, the proportion of the nonbilayer lipid MGDG decreases with a concomitant increase in bilayer-forming lipids (SQDG) (Fig. 1). Altogether, these changes contribute to membrane stabilization at high temperatures [34, 35, 42]. In Cyan10605, the amount of total TAG, in particular fully saturated molecular species (46:0, 48:0), was increased at high temperatures, reflecting the increase in 16:0 in the membrane lipids (Figs. 1c and 2a). These results show that the TAG pool is in exchange with acyl groups from membrane lipids for which it presumably serves as a reservoir. The amounts of the photosynthesis-related nonpolar lipids, FAPE, acyl-PQH and acyl-PQC were decreased, presumably to provide substrates (phytol, plastoquinol) in line with the general decrease in photosynthetic membranes at high temperatures (Figs. 1b and 2b-d) [43].

### Lipid changes in Cyan10605 during growth in seawater medium

While *C. aponinum* can grow in seawater in the presence of 34 g L^− 1^ of NaCl equivalent to the average salt concentration in the surface water of the oceans, we selected ASN-III medium with a NaCl content of 25 g L^− 1^ [6]. In addition to containing relatively high amounts of NaCl, the ASN-III medium which mimics the natural seawater in the oceans, differs from BG-11 due to higher levels of calcium (CaCl_2_) and magnesium (MgSO_4_, MgCl_2_) and lower concentrations of nitrogen (NaNO_3_) and phosphorus (K_2_HPO_4_). Growth of Cyan10605 cells was strongly suppressed in ASN-III medium (Fig. 3a). In seawater medium, fimbriae-like protrusions occurred on the cell surface which might be involved in supporting motility, adhesion, or biofilm formation (Fig. 9d) [44, 45]. The accumulation of carboxysomes (Fig. 11d) can be explained by the fact that the solubility of CO_2_ is reduced with increased salinity in water [46]. Therefore, the increased abundance of carboxysomes enhances the affinity of RuBisCO to CO_2_ and helps to maintain carbon fixation. In seawater medium, the cells accumulated large amounts (50 mol%) of the sulfolipid SQDG at the expense of galactolipids (Fig. 3d, e). Cultivation of the freshwater cyanobacterium *Synechococcus* PCC 6311 with 30 g L^− 1^ NaCl caused membrane lipid remodeling associated with a shift from MGDG to DGDG, SQDG, and PG. The decrease of the MGDG/DGDG ratio and the accumulation of the anionic lipids (SQDG, PG) might enable the cells to tolerate the effect of high Na^+^ concentration on the cell surface [47]. Growth of Cyan10605 in high salt medium presumably requires the reinforcement of the membranes with an anionic lipid, such as SQDG (Fig. 3d, e) to withstand osmotic pressure and prevent the influx of NaCl or efflux of water. PG, the only other anionic lipid in Cyan10605 membranes, cannot be synthesized under the low phosphate conditions of the seawater medium. The amounts of all nonpolar lipids decreased in seawater medium (Fig. 4a-d), presumably reflecting the requirement of acyl groups, phytol and plastoquinol to sustain the synthesis of thylakoid membranes and the photosynthetic apparatus.

### Lipid responses of Cyan10605 to phosphate deprivation

Phosphate limitation prevents the synthesis of ATP, nucleic acids and phospholipids, which together constrain cell growth. The growth arrest on day 3 for phosphate depleted cells is due to the lack of phosphate (0 g L^− 1^). This was confirmed by nutrient analysis of the culture supernatant using ICP-OES, which showed that phosphate remained undetectable at all time points under -P conditions (Fig. S1). In contrast, in phosphate-replete cultures, cells continued to grow beyond day 3, consistent with the presence of phosphate in the medium (> 2 mg L⁻¹) and the occurrence of polyphosphate bodies at day 7 (Fig. 9f; Fig. S1a). ICP-OES analysis revealed that several trace elements, including Fe, Zn, and Mn, were strongly depleted during cultivation, particularly under phosphate-replete conditions, suggesting that the limitation of these micronutrients might contribute to growth arrest in the stationary phase (Fig. S1a). In contrast, concentrations of S, Mo, B, Cu, Na, K, and Co remained largely unchanged, while Mg and Ca levels, although declining, remained above concentrations likely to limit growth. Elemental analysis of total nitrogen and carbon in the culture supernatant further indicated that nitrogen and total carbon (predominantly present as non-volatile inorganic carbon, e.g., carbonate/bicarbonate) were not depleted under these conditions. As bicarbonate and carbonate serve as sources of CO₂ for photosynthesis, this supports the conclusion that growth limitation in phosphate-replete cultures was not caused by nitrogen or carbon limitation, but more likely by depletion of micronutrients such as Fe, Zn, and Mn (Fig. 1b).

Cyan10605 cells growing under phosphate starvation revealed a pronounced fragmentation of thylakoids and a more distinct phycobilisome structure (Fig. 11f), together with a decrease in chlorophyll content (Fig. 5b) [48]. Under low phosphate conditions, the cells were completely devoid of polyphosphate bodies used as phosphate storage (Fig. 11f) [49]. Cyanophycin granules accumulated in Cyan10605 under phosphate deprivation (Fig. 11f), and similarly in *Synechocystis* sp. PCC 6803 and *Nostoc* sp. PCC 7118 [48, 50]. This suggests that Cyan10605 retained the nitrogen and/or carbon released from the proteins degraded during phosphate starvation in the storage compound cyanophycin (a polymer of aspartic acid and arginine) [48, 50]. In *Synechococcus* sp. PCC 7942 and *Synechocystis* sp. PCC 6803, DGDG and SQDG increased at the expense of PG and MGDG under phosphate deprivation [2, 51]. We also observed a decrease in MGDG and an increase in the proportion of DGDG, while the proportions of SQDG and PG remained similar during growth under phosphate deprivation (Fig. 5e). The PG content in Cyan10605 grown in modified BG-11 medium is unusually low with only 2.0 mol%, and it was further reduced to 1.2 mol% after phosphate deprivation (Fig. 5e). The low PG content is rarely found among cyanobacteria because MGDG, DGDG, SQDG, and PG constitute 40–60%, 10–20%, 10–20%, and 10% mol, respectively in most cyanobacteria [34, 35, 52]. In *Synechococcus* sp. PCC 7942, the amounts of SQDG and PG were 10 and 16 mol% (at 0.04 g L^− 1^ phosphate), 22 and 7 mol% (0.004 g L^− 1^ phosphate) and 18 and 3 mol% (0 g L^− 1^), respectively [2, 34, 35, 53]. Sulfolipid appears to be the preferred anionic lipid in Cyan10605 compared to PG, as the SQDG content is higher than that of PG under all selected growth conditions, and it increases to extremely high amounts (~ 50 mol%) in seawater medium (Fig. 3e). Phytoplankton adapts to phosphorus limitation by remodeling membrane lipids, replacing phospholipids with non-phosphorous lipids such as SQDG and betaine lipids [34, 35, 54]. Cyan10605 is not the only cyanobacterium with a high SQDG to PG ratio of ~ 10 under phosphate-replete conditions. In *Prochlorococcus* sp. MED4 which is a member of the phytoplankton, the SQDG/PG ratio is ~ 20 under phosphate-replete conditions [3]. The high SQDG content in Cyan10605 might be due to the adaptation to the extreme environment of Euganean thermal springs from which it originates [5]. The increased SQDG content in Cyan10605 is presumably due to high expression of the sulfolipid synthase gene *sqdB* under phosphate replete conditions [39]. SQDG in Cyan10605 contained a high proportion of molecular species with two C16 fatty acids (16:0–16:0, 16:0–16:1), compared to other membrane lipids which are mostly composed of 14:0–16:1, 14:1–16:0, and 14:0–16:0 species (Figs. 1, 3, 5, 7 and 9). These results are in line with the high C16 fatty acid content of SQDG in other cyanobacteria like *Cyanothece* sp. PCC 8801, while 14:0 is the most abundant acyl group in the other membrane lipids of *Cyanothece* [35]. The SQDG-deficient *sqdB* mutant of *T. elongatus* BP-1 showed severely impaired growth under phosphate deprivation, concomitant with an inhibition in PSII activity, which could not be fully rescued by PG supplementation, demonstrating the importance of SQDG for maintaining photosynthesis during phosphate deprivation [39]. The TAG content in Cyan10605 remained similar, but the amount of FAPE increased to nearly 100 pmol mg^− 1^ DW during phosphate deprivation, presumably reflecting the uptake of phytol derived from chlorophyll degradation (Figs. 5b and 6b) [55]. The amounts of acylated plastoquinols were decreased (Fig. 6c, d), likely to provide plastoquinol for the photosynthetic apparatus under phosphate deprivation.

### Lipid responses to N deprivation

Under nitrogen deprivation, growth of Cyan10605 was severely inhibited and the cells showed a pale green color due to the loss of chlorophyll (Fig. 7a, b). Nitrogen starvation stimulates TAG production in green algae like *Chlamydomonas reinhardtii* [56]. In many cyanobacteria including *Synechocystis* sp. PCC 6803, however, light-harvesting complexes and thylakoid membranes are degraded, and carbon is deposited in the form of glycogen and polyhydroxybutyrate under nitrogen deficient conditions [57]. After growth under nitrogen deprivation for 5 days, the amounts of MGDG, DGDG and PG were decreased in Cyan10605, but that of SQDG remained similar, resulting in the increased proportion of SQDG (Fig. 7d, e). Therefore, SQDG is the preferred anionic membrane lipid in Cyan10605 that remains at a high proportion during stress, including high temperature, seawater medium and nitrogen deprivation (Figs. 1, 3 and 7). The decomposition of thylakoid membranes was confirmed by electron microscopy which also showed a drastic accumulation of carboxysomes under nitrogen deprivation (Fig. 11h). The increase in carboxysomes might be explained by the increase in RuBisCO which can serve as a nitrogen reservoir [58, 59]. Nitrogen deprivation resulted in an accumulation of TAG which was increased by 46% in Cyan10605. Notably, most of the accumulated TAG species contained at least one C18 acyl group (C50, C52, C54 TAG) (Fig. 8a). Furthermore, N deprivation caused a shift from medium chain fatty acids (C14) to long chain fatty acids (C18) in membrane lipids, represented by PG, in which 34:2 (16:1–18:1), 34:1 (16:0–18:1, 16:1–18:0), and 36:2 (18:1–18:1) species were unusually high (Fig. 7c, I). C18 fatty acids are unusual for Cyan10605, as they do not accumulate in other membrane lipids or under the other growth conditions tested in this study. Under -N conditions, acyl groups in TAG presumably cannot be directly derived from membrane lipid breakdown, but they are proposed to be elongated through one or two rounds of fatty acid elongation reactions of C14 and C16 fatty acids. The amount of FAPE was decreased in Cyan10605 during N deprivation, presumably caused by chlorosis which led to a degradation of chlorophyll and its side chain phytol (Figs. 7b and 8b) [60]. Both plastoquinol esters showed a drastic decrease in the amount (Fig. 8c, d). The declined level in acylated plastoquinols might be due to the degradation of photosynthetic membranes and apparatus which is accompanied with the loss of plastoquinol (Fig. 11h).

### Lipid changes of Cyan10605 cells during extended cultivation

To study the adaptation of Cyan10605 cells to continuous growth in a batch culture, we recorded the lipid changes after 42 days of extended cultivation, during which the cultures turned from blue-green to pale yellow (chlorosis) due to the nearly complete loss of chlorophyll (Fig. 9a). This chlorosis effect was similar to the changes observed during nitrogen deprivation (Figs. 7b and 9a). The loss of chlorophyll was also observed by light microscopy, because 14 d-old cells were green, while 42 d-old cells were pale (Fig. S2). The 14 d and 42 d-old cells could not be stained with the non-penetrating dye Evans Blue [27, 28]. Only after boiling the cells, they were stained with Evans Blue (Fig. S2). These results indicate that the cells were still viable after 42 d of incubation. Under extended cultivation in a batch experiment (42 days), depletion of Fe, Zn, and Mn was already evident after 14 days as determined by ICP-OES measurements. Therefore, limitation of these micronutrients might become a major factor affecting growth and metabolism during prolonged cultivation (Fig. S1c) [61, 62]. Similar to the growth experiments in the presence or absence of phosphate, total nitrogen and total carbon remained largely unchanged after 28 and 42 days of cultivation, suggesting that they were not limiting for growth.

The amounts of TAG and FAPE were increased, with TAG reaching ~ 250 pmol mg^− 1^ DW which was the highest amount observed across all tested conditions (Fig. 10a, b). The accumulation of TAG and FAPE during extended cultivation is presumably caused by the incorporation of acyl groups and phytol released during membrane lipid and chlorophyll degradation (Fig. 9b, c) in Cyan10605 during extended growth similar to plants [63, 64]. At the same time, the amounts of acyl-PQH and acyl-PQC were strongly decreased (Fig. 10c, d). The decrease in acylated plastoquinols might be caused by the degradation of photosynthetic membranes as indicated by electron microscopy (Fig. 11j). This result shows that the synthesis of TAG, FAPE and acylated plastoquinols which are presumably derived from the MFAT activity, can be differentially regulated under chlorotic growth conditions (Figs. 10 and 12) [22].

### The multifunctional acyltransferase CyanMFAT is involved in nonpolar lipid synthesis

We previously showed that the MFATs from *Synechocystis* sp. PCC 6803 (Slr2103) and *Synechococcus* sp. PCC 7002 (A0918) harbors acyltransferase activities for the synthesis of TAG, FAPE, acyl-PQH and acyl-PQC [22]. After expression in *E. coli*, we demonstrate here that Cyan10605_2185, the only MFAT found in Cyan10605, can synthesize TAG and FAPE (Fig. 10), but we could not demonstrate acyltransferase activity with the artificial substrate decylplastoquinone. It was recently shown that the lipid droplets of cyanobacteria (‘cyanoglobules’) contain the nonpolar lipids TAG, acyl-PQH and acyl-PQC [65]. While the absolute amounts of acyl-PQH and acyl-PQC could not be determined due to the lack of internal standards, it is likely that their decrease under all growth conditions contributes to the disappearance of the lipid droplets. Functional studies in Cyan10605 deletion mutants were not possible due to the difficulties in transformation. This can be explained by the abundance of restriction/modification and CRISPR systems in *C. aponinum*, which may serve as a barrier to the introduction of foreign DNA [9, 66]. Therefore, further studies are required to decipher the functional roles of the MFAT and specific neutral lipids during stress adaptation in Cyan10605.

## Supplementary Information

Supplementary Material 1

## Data Availability

All data are included in the manuscript. Other data that support the findings of this study and the corresponding materials are available from the corresponding author upon reasonable request.
