# Supplementary material for: Adaptation of lipid metabolism of the polyextremophilic cyanobacterium Cyanobacterium aponinum PCC 10605 to adverse environmental conditions
Source: BMC Plant Biol. 2026 Apr 22;26:734. doi: 10.1186/s12870-026-08797-z (PMC13101131; doi:10.1186/s12870-026-08797-z)
Supplement: Supplementary file 1 — Supplementary Material 1 [file 12870_2026_8797_MOESM1_ESM.pdf]

Fig. S1a, b

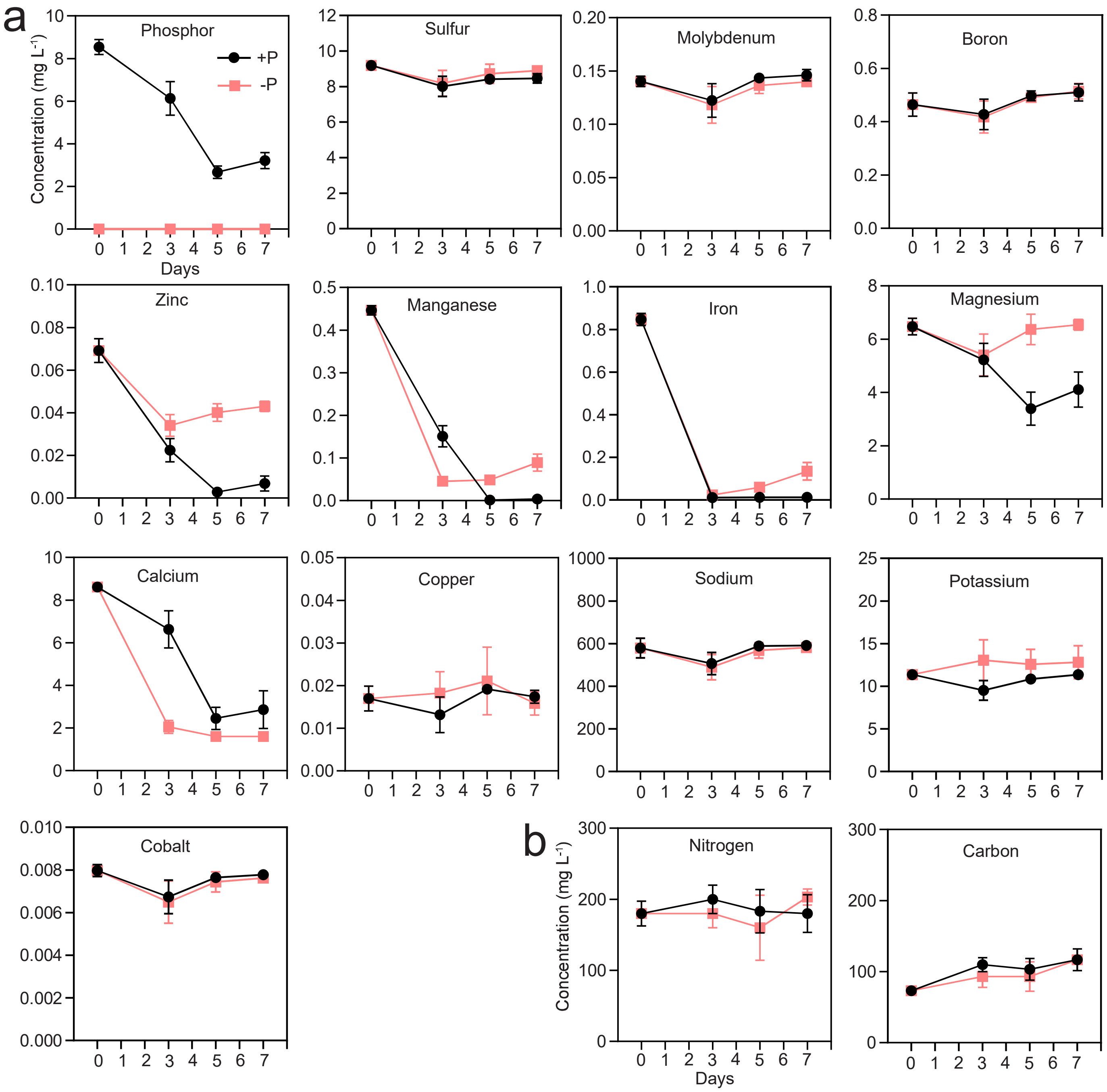

a
b
Phosphor
Sulfur
Molybdenum
Boron
Zinc
Manganese
Iron
Magnesium
Calcium
Copper
Sodium
Potassium
Cobalt
Nitrogen
Carbon
+P
-P
Concentration (mg L-1)
Days

Fig. S1c, d

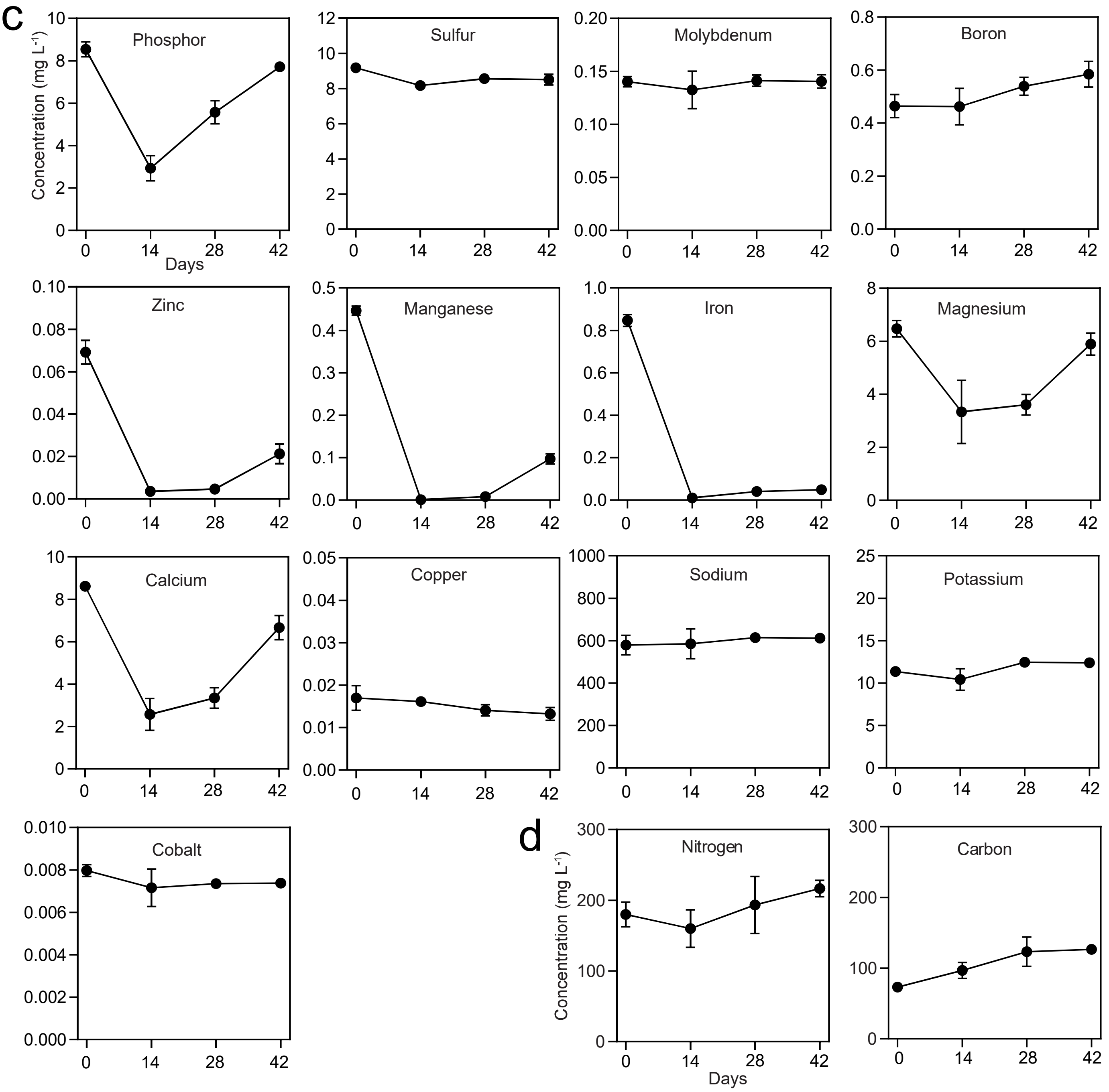

Fig. S1 Nutrient depletion in BG-11 medium during phosphate deprivation and extended cultivation. (a, b) Cyan10605 cells grown under phosphate-replete and phosphate-depleted conditions for 7 days. (c, d) Cyan10605 cells grown in modified BG-11 medium for 42 d (extended cultivation). Samples of the culture supernatant were centrifuged to remove the cells. The elements P, S, Mo, B, Zn, Mn, Fe, Mg, Ca, Cu, Na, K and Co were measured by ICP-OES (a, c). Total nitrogen (N) and total carbon (C) were measured by elemental analysis (gas chromatography) (b, d). N=3-5; means ± SD.

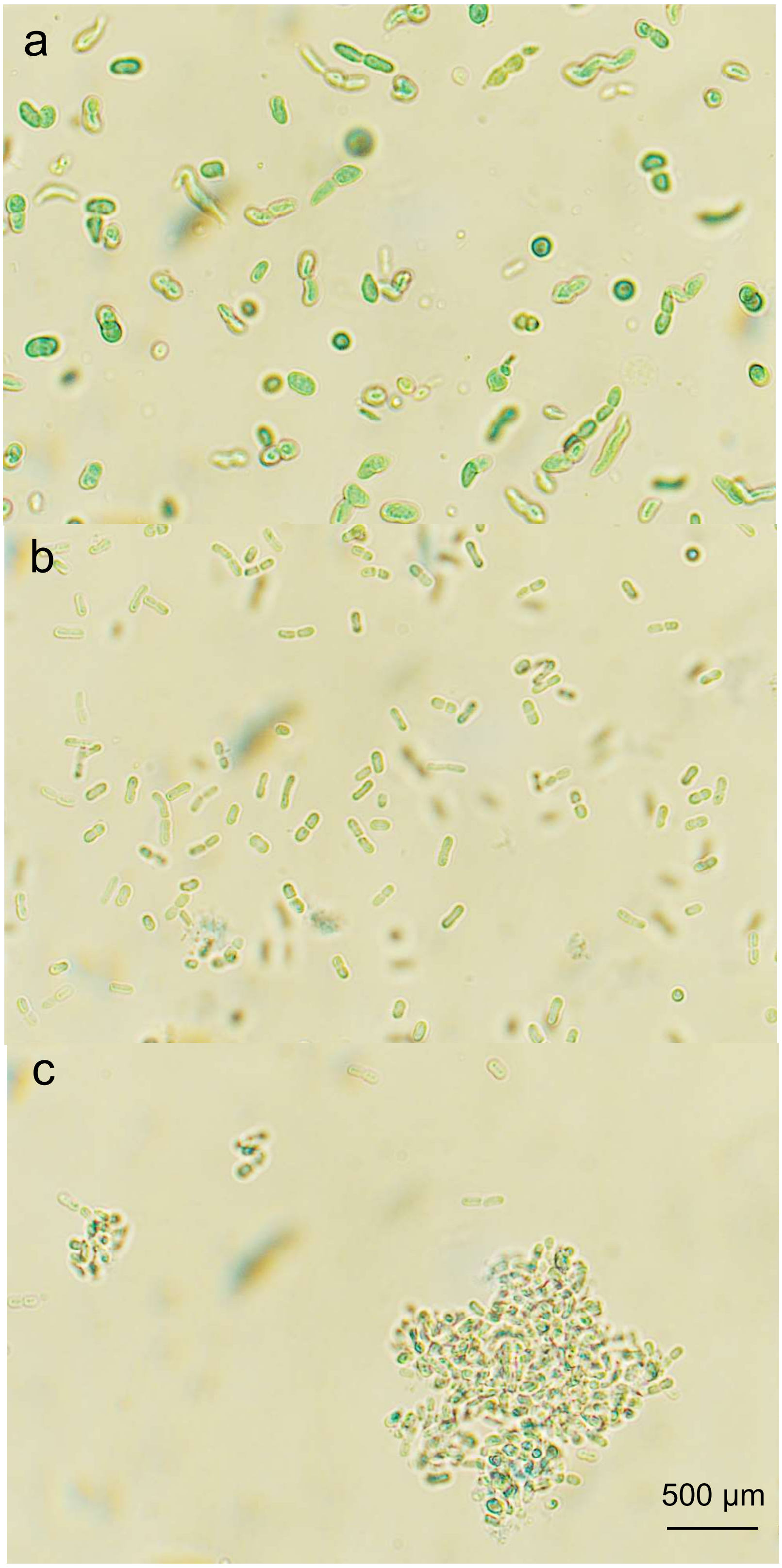

Fig. S2 Viability of Cyan10605 cells after extended cultivation Cyan10605 cells were grown for 14 or 42 d in modified BG-11 medium. Cells were incubated with Evans Blue, washed and observed by light microscopy. (a) Cyan10605 cells grown for 14 d are green (chlorophyll) but are not stained with Evans Blue. (b) Cyan10605 cells grown for 42 d are smaller, rod shaped, and contain less chlorophyll. The cells are not stained with Evans Blue. (c) Cyan10605 cells grown for 42 d were boiled for 20 min. Several dead cells are stained with Evans Blue.

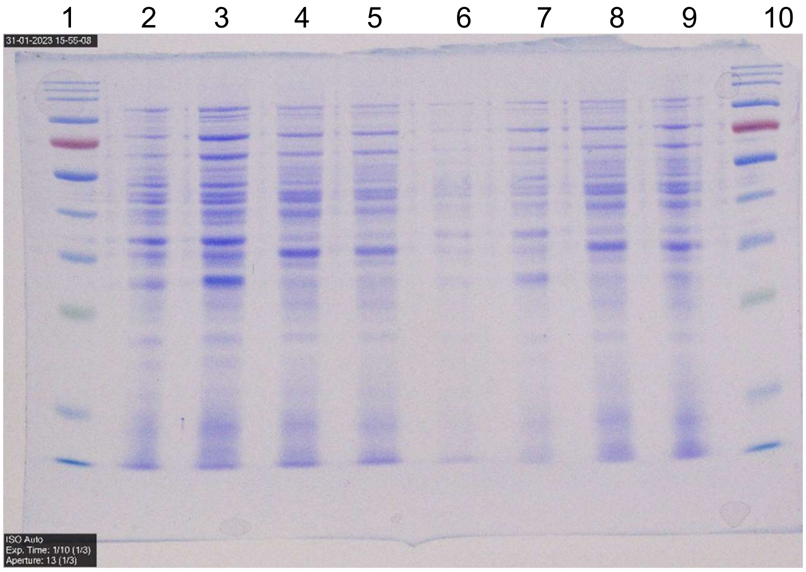

SDS-PAGE of pQE80L-Cyan10605 (MFAT) 27.01.2023
Stained with Coomassie Brilliant Blue
Lanes 1, 10: protein ladder (10-250 kDa)
2, 6: pQE80L-EV (E. shox) #1, #2 3, 7: pQE80L-CapTAG (MFAT) (E. shox) #1, #2
4, 8: pQE80L-EV (BL-21, AI) #1, #2 5, 9: pQE80L-CapTAG (BL-21, AI) #1, #2

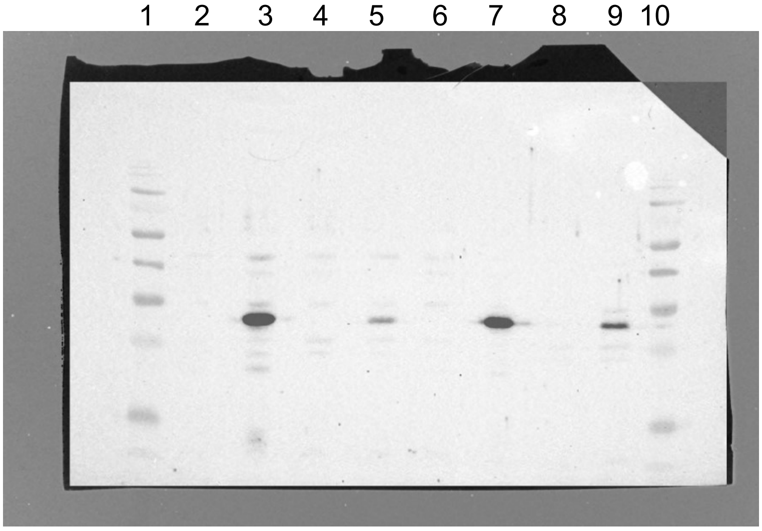

Western blot of pQE80L-CapTAG (MFAT) 27.01.2023
Lanes 1, 10: protein ladder (10-250 kDa)
2, 6: pQE80L-EV (E. shox) #1, #2 3, 7: pQE80L-CapTAG (MFAT) (E. shox) #1, #2
4, 8: pQE80L-EV (BL-21, AI) #1, #2 5, 9: pQE80L-CapTAG (MFAT) (BL-21, AI) #1, #2
After Western blot, His-tag proteins identified using HisDetector Nickel-HRP (Kirkegaard & Perry Lab.), and visualized by chemiluminescence using Pierce ECL Plus Western Blotting Substrate (ThermoFisher Scientific).

Fig. S3 Original photos of the protein gel and western blot for Figure 12.
